# Patient complaints in dentistry: A qualitative meta-synthesis

**DOI:** 10.1371/journal.pone.0362563

**Published:** 2026-11-12

**Authors:** Lorraine Snyman, Thomas Corne Postma, Werdie Van Staden

**Affiliations:** 1 Department of Dental Management Sciences, Faculty of Health Sciences, University of Pretoria, Pretoria, South Africa; 2 Centre for Ethics and Philosophy, Faculty of Health Sciences, University of Pretoria, Pretoria, South Africa; UCLA School of Dentistry, UNITED STATES OF AMERICA

## Abstract

**Introduction:**

Patient complaints in dentistry concern their dissatisfaction, safety concerns, quality of care, and challenges within the patient-practitioner relationship. Whilst existing research of these complaints has largely focused on quantitative analysis of regulatory complaints and malpractice, these studies capture little of the meaning that patients express in their complaints. This study synthesized a qualitative understanding of patient complaints across various dentistry settings.

**Method:**

This qualitative meta-synthesis followed the six-step approach of Sandelowski and Barroso, drawing on previously published qualitative findings from various studies that constituted a higher-order case as suggested by Hoon’s design framework. Data was sourced through a comprehensive search of health databases (MEDLINE, PsycINFO, PubMed, Scopus, Web of Science, CINAHL). From studies found suitable upon appraisal, findings were subjected to reflexive thematic analysis. The resulting synthesis is reported according to the PRISMA guidelines extended for qualitative evidence synthesis.

**Results:**

From an initial 857 studies, 19 met criteria for inclusion. Complaints reflected patient experiences in four inter-related domains of concern, namely, 1) impeded access to dental care, 2) unsatisfactory communication, interactions and attitudes, 3) deficient clinical treatment, and 4) unethical and unprofessional conduct. These domains may be understood as underlying processes in which patients experienced threats to trust, patient agency and autonomy, relational security, and that professional and ethical obligations have been breached. They may also be understood as signals of broader systemic and relational functioning.

**Conclusion:**

Our findings suggest that complaints may reflect breakdowns in the fiduciary patient-practitioner relationship and constitute an important source of insight into the relational and systemic conditions necessary for trustworthy, person-centered dental care.

**Clinical relevance:**

The four domains yielded in this synthesis may inform dentists and health systems on the scope of compromised relationships and systemic deficiencies as well as efforts to improve the quality of dental care and reduce patient complaints.

## Introduction

Some dental practices, particularly in private sectors, have adopted commercial and consumer-oriented business models, generating ethical tensions as practitioners balance financial imperatives with professional duties. The commodification of dental services risks compromising the fiduciary nature of the patient-practitioner relationship, turning care into a transactional interaction rather than a therapeutic alliance [1]. The tension between commercial pressures and ethical duties often manifests as patient dissatisfaction and formal complaints, especially when care is viewed as profit-driven or access is restricted due to systemic limitations [2]. Simultaneously, a substantial portion of the population remains dependent on public healthcare systems, where access to even basic dental services is often limited. These disparities exacerbate existing health inequities and place additional ethical burdens on practitioners working within resource-constrained environments [3].

Patient complaints in dentistry, therefore, represent a significant ethical and professional challenge for both dental practitioners and healthcare systems. An Australian study reported that dentists risk being disproportionately subject to complaints compared with other healthcare professionals, owing to the unique nature of dental care delivery [4]. Dental treatment often begins with invasive procedures from the first consultation, may extend over multiple sessions, and is frequently associated with pain, anxiety and significant financial costs, all of which heighten patient sensitivity and expectations. When expectations of quality treatment, compassion, and transparency are unmet – whether due to clinical outcomes, communication failures, or systemic limitations – complaints arise, often reflecting deeper concerns about access, safety, trust and the patient-practitioner relationship [5–9]. Effective complaint management is therefore essential, not only for maintaining trust but also for informing service improvements and enhancing patient satisfaction [10].

Research on patient complaints is recognized within healthcare systems and regulatory bodies, for gauging the extent of patient dissatisfaction, and to provide actionable insights into safety, quality of care, and organizational accountability. While attention to this field is shaped by regulatory requirements and public expectations, its relevance lies in the ability to complement traditional performance metrics with patient-centered evidence [11–13]. Existing dental complaint research has largely focused on quantitative analysis of regulatory complaints and malpractice claims [4,14–23]. Whilst such studies identify the frequency and characteristics of complaints, they provide little insight into the meaning patients express in their experiences. Patient narratives can offer a unique perspective on aspects of care that patients perceive as harmful, inadequate or ethically troubling. Qualitative studies have begun to illuminate patients’ experiences and interpretations of dissatisfaction and complaint, but this evidence remains dispersed across different healthcare settings, complaint sources and areas of concern. To date, no known qualitative meta-synthesis has brought together this body of evidence to examine the shared meanings and ethical significance that patients attach to complaints about dental care. Consequently, there is a gap in understanding how individual accounts of concern may converge to reveal broader concerns about the patient-practitioner relationship. A qualitative meta-synthesis provides an opportunity to move beyond describing the prevalence or categories of complaints towards an interpretive account of what patients’ complaints signify across diverse dental settings [24].. By synthesizing findings across qualitative studies, the present study seeks to identify patterns of meaning that may not be apparent within individual studies and to interpret these patterns through a relational-ethical lens. Such an approach may contribute to understanding patient complaints not merely as expressions of dissatisfaction, but as accounts of perceived failures in professional and relational obligations. The aim of our study was therefore to synthesize and interpret the meanings expressed in qualitative accounts of patient complaints about dental care across various settings, and to develop a higher-order understanding of their ethical and relational significance

## Method

### Design

We conducted a meta-synthesis following the six-step guideline proposed by Sandelowski and Barroso [25] for synthesizing qualitative research. In this approach, findings from primary qualitative studies are integrated through an interpretive and analytical process to generate new conceptual understandings of a phenomenon. Qualitative meta-synthesis refers both to the interpretive product and the analytical process through which findings are combined, integrated, and reinterpreted across studies [25]. The six steps of the process involved conceiving the qualitative research synthesis study, searching for and retrieving study reports, appraising these study reports, classifying the findings, synthesizing findings in a meta-synthesis and synthesizing findings in a meta-summary [25]. The synthesis was further informed by Hoon’s [26] conceptualization of qualitative synthesis as a higher-order case study. Within this framework, the corpus of included studies was treated as the bounded higher-order case, while each included study represented an embedded subcase. Collectively, these subcases provided contextualized accounts of patient complaints in dentistry across diverse settings and sources. This perspective supported an interpretive synthesis aimed at identifying recurring meanings, experiences, and explanatory patterns across studies using different qualitative approaches, settings and data sources [26]. The higher-order case was bounded conceptually by the overarching phenomenon of interest, namely qualitative meanings and experiences embedded in formally documented dental complaints, which represented, irrespective of settings, formalized expressions of perceived failures of care. Variation across settings was treated as contextual nuance within the same overarching phenomenon. This allowed the synthesis to examine whether meanings recurred across contextual differences while retaining attention to the circumstances in which those meanings arose. We published a protocol in the International Prospective Register of Systematic Reviews (PROSPERO ID nr CRD420251052650) and followed the PRISMA guidelines [27] for reporting the synthesis (Checklist available in S1 Appendix). We obtained ethics approval for the study from the Faculty of Health Sciences Research Ethics Committee of the University of Pretoria (Ethics Reference Number 313/2024).

### Inclusion and exclusion criteria

The study included peer-reviewed articles in English that focused on formally documented patient complaints in dentistry, primarily utilizing qualitative research designs. Owing to language proficiency constraints within the research team, only English-language publications were considered. We considered mixed-method studies only if they separated their qualitative and quantitative components, allowing for the analysis of only the qualitative data. This approach was adopted after the initial exploration had revealed that pure qualitative studies in this area were limited. To be eligible for inclusion mixed-method studies should therefore had a distinct qualitative methods description (e.g., thematic analysis, qualitative content analysis), clearly distinguishable qualitative findings or results and supporting qualitative material such as participant quotations or clearly labelled author-interpreted themes grounded in qualitative analysis.

The following exclusion criteria applied:

- Articles that concentrate on complaints about or dissatisfaction with specific dental procedures (e.g., implants, dentures, amalgam restorations, etc.) instead of addressing general care issues. This refers to articles where patients complain about technical aspects of the treatment modality, and not about the care itself.
- Articles that did not express formal complaints about dental care
- Articles that dealt exclusively with the complaint process.

### Search strategy and study selection

We used the SPIDER search tool [28] to identify index terms for conducting the literature search (Sample, Phenomenon of Interest, Design, Evaluation, Research type). We deemed it acceptable if the participant sample consisted of either patients or their family members, with the focus being on patient grievances. The emphasis was placed on qualitative research and qualitative analysis methods.

We conducted a search of health databases (MEDLINE, PsycINFO, PubMed, Scopus, Web of Science, CINAHL) in September 2024 and updated the search on 26 August 2026. The following MeSH terms and keywords were used: ((malpractice* or dissatisfaction* or negligenc* or Dissatisfied) and (complain* or grievanc* or claim* or “patient advocacy” or Disclosure) AND (dent* or dental or dentist or dentistry or Oral Health or periodontist or Orthodontist or endodontist or maxillo-facial surgeon) AND (qualitative or interview* or phenomenolog* or experience* or themes or thematic or narrat* or hermeneutic* or “grounded theor*” or “focus group” or audiorecording* or “audio-recording*” or ethnograph* or “content analys*”). No restriction was placed on the publication date of articles. A targeted keyword search of Google Scholar was also conducted, supplemented by hand searching reference lists of relevant articles. Results of searches were exported into Endnote^TM^ and then transferred into Covidence systematic review software, by Veritas Health Innovation, Melbourne, Australia 2025 (available at www.covidence.org). After all the duplicates were removed, two reviewers (CP and LS) independently screened the titles and abstracts of the articles for relevance, and disagreements were resolved through discussion between the reviewers. An audit trail of article screening was kept in Covidence which also calculated the reviewer agreement. The full texts of relevant studies were retrieved and then read and reviewed independently by the two reviewers according to the inclusion criteria.

### Quality appraisal

We used the Joanna Briggs Institute Qualitative Appraisal and Review Instrument (JBI-QARI) [29] to assess the eligible articles for methodological validity. The JBI-QARI was chosen because research suggests that it is a coherent appraisal tool orientated towards congruity [30]. The JBI-QARI instrument consists of 10 questions that guide the evaluation focus to the congruity between the research methodology and the stated philosophical perspective, the research question or objective, the methods for data collection, the data representation and analysis, and the data interpretation; a statement locating the researcher culturally or theoretical, the influence of the researcher on the research, representation of participants’ voices, evidence of ethical approval and on the conclusions drawn in the report. The appraisal was done independently by two reviewers (CP and LS) and conflicts were discussed until consensus was reached. The appraisal assisted the researchers in gaining insight into the differences between studies and to appreciate the limitations and strengths. In line with Sandelowski and Barroso’s advice, we decided that no articles will be excluded based on their quality, as this might result in the exclusion of reports with findings that might still be valuable to practice [31].

### Data extraction

We extracted the key characteristics of the eligible articles into a table and included information on the authors, the country where the study took place, aim, setting, sample, research design, and key findings. Presenting the key characteristics in a table format will enable readers to independently draw conclusions about the included material. The above-mentioned characteristics were extracted separately from the qualitative findings that constituted the synthesis dataset.

### Analysis and synthesis of findings

Two researchers were involved in the analysis and synthesis. The first reviewer (LS) imported the full-text articles into Atlas.ti 25 for Windows, to code data and manage the qualitative analysis. We used reflexive thematic analysis as described by Braun and Clarke [32,33] as the analytic approach for identifying patterns of meaning across the qualitative findings of the included studies. We defined clear boundaries to ensure consistency in what was treated as data across studies. For each article, we restricted analysis to the sections labelled as “Results” or “Findings” and any equivalent sections that presented author-interpreted themes and/or illustrative participant quotations. In mixed-method studies, qualitative findings were distinguished from quantitative results and only the qualitative results were coded. Descriptive statistics, numerical summaries, and quantitative complaint categories were explicitly excluded to ensure that pre-existing frameworks did not bias our inductive generation of codes.

The analysis began with LS reading the results section of each article line by line to become familiar with the data. LS then inductively coded all the qualitative information contained in the results sections, including direct quotations, focusing on what each extract indicated about the nature and meaning of the complaint. Inductive coding was complemented by deductive attention to the research question, in the sense that emergent codes were continually checked for their relevance to patients’ complaints about dental care and refined or discarded when they did not contribute meaningfully to this focus [32]. We employed both semantic and latent coding. Semantic coding was applied initially to capture the explicit, surface-level meanings of the data, ensuring the synthesis remained firmly grounded in the patients’ reported experiences [32]. Subsequently, latent coding was used to interpret the underlying relational and ethical meanings connecting these surface-level complaints [32]. For example, descriptions about poor scheduling were initially coded semantically as administrative inefficiencies; through latent interpretation, they were considered alongside findings on poor availability and responsiveness and ultimately understood as representing an overarching issue of “impeded access to care.” In this way our coding moved beyond surface-level description towards explaining how disparate grievances could reflect common underlying concerns.

The second reviewer (CP) independently reviewed and interrogated LS’s coding outputs. Differences in interpretation were discussed reflexively, returning to the underlying qualitative extracts and considering alternative readings. Where disagreement arose, interpretations were retained, modified or discarded according to their grounding in the qualitative material, conceptual coherence and explanatory contribution. Analytic discussions and decisions were documented in reflexive notes.

Following the initial coding, LS and CP independently compared related codes within and across studies, organizing them into conceptually related meaning clusters based on their similarities, differences and relationships. From the outset we organized these clusters based on their “analytical emphasis” rather than seeking clear-cut, mutually exclusive boundaries, as overlap in patient experiences was expected [32,33]. We then engaged in repeated discussions to refine these meaning clusters into candidate themes and subthemes. At this stage, we examined whether groups of codes formed coherent patterns of meaning across the dataset and whether they offered explanatory insight beyond descriptive complaint categories and therefore accommodated both convergent and divergent accounts [32]. For example, codes related to long waiting times, difficulty obtaining appointments and being turned away from care were not only grouped descriptively as “access problems” but were interpreted as signaling perceived disregard for patients’ needs and priorities. Recurrence across studies was considered as evidence of cross-case relevance but was not treated as a prerequisite for analytic significance [32].

Theme development was iterative, involving continual movement between original findings, codes, meaning clusters and themes [32,33]. As codes were compared across studies, we considered both their substantive content and the primary concern expressed by the complaint. Where similar content could be located within more than one domain, we used the main analytical emphasis of the complaint to determine its principal coding location. Thus, theme boundaries were based not simply on the presence of particular topics (e.g., communication, treatment outcome), but on what the complaint was principally understood to signify within the reported experience. When a code appeared to fit more than one theme, it was reviewed against the developing theme definition and either retained in the theme that best reflected participants’ emphasis, or the themes were reformulated more precisely. For example, complaints about treatment outcome could relate to either deficient clinical treatment or to unethical and unprofessional conduct. These were distinguished by their underlying concern: poor treatment, complications or poor aftercare were situated within deficient clinical treatment, whereas allegations of concealment of errors or inappropriate refusal of care were situated within unethical and unprofessional conduct. This reflexive and collaborative process was repeated until we were confident that the final thematic structure provided a coherent account of the research question while remaining responsive to contextual differences between studies [32].

## Results

### Study selection

The PRISMA flow diagram, illustrating the literature search and screening process, is shown in Figure 1. After identifying 857 studies through our database searches, of which 29were retrieved for full text review, we included 19 studies in the review. The initial agreement between reviewers, before discussion, for the title and abstract screening and full text screening were 97% (Cohen kappa 0.47) and 93% (Cohen kappa 0.85), respectively.

**Fig 1:**
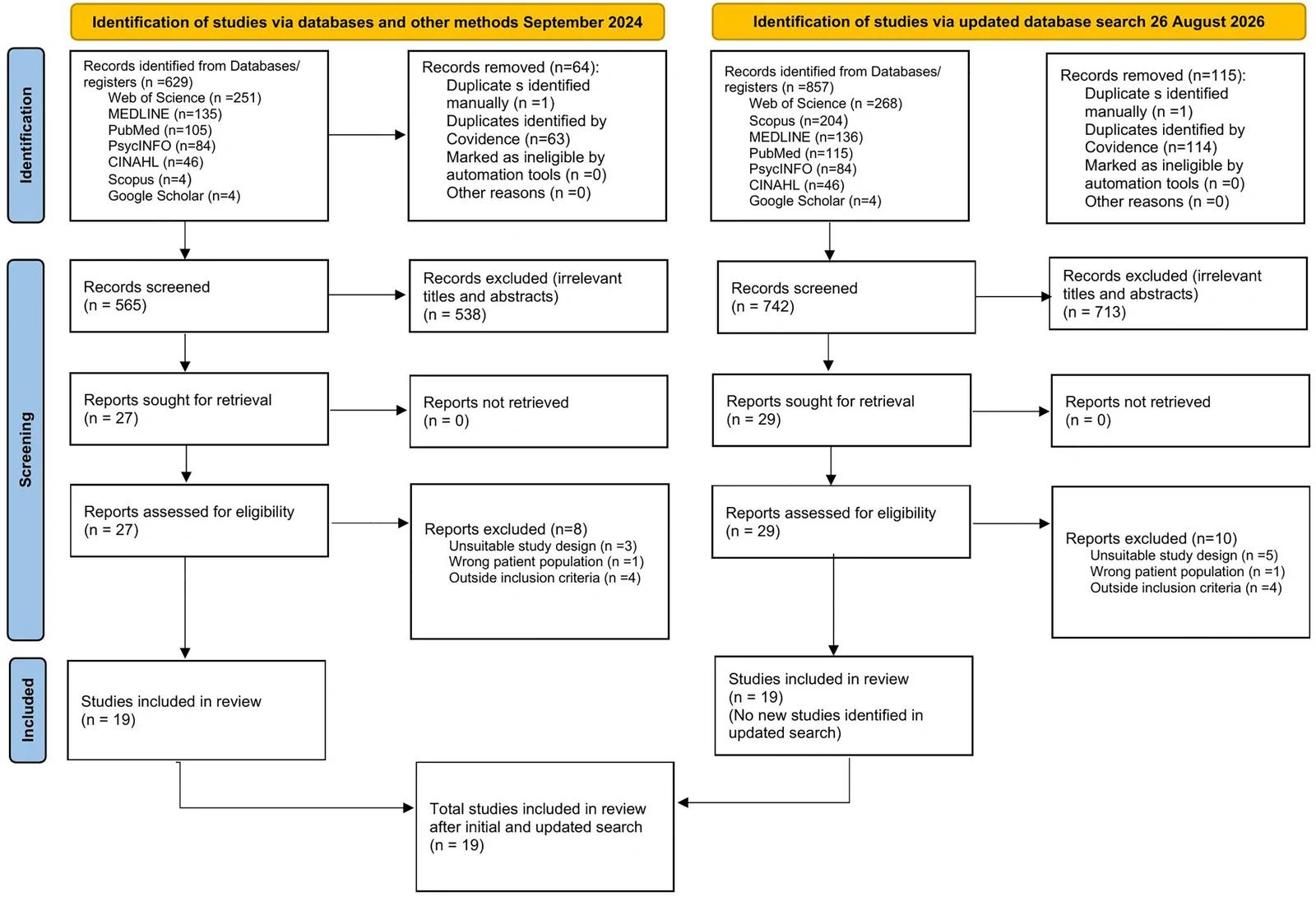
PRISMA flow diagram illustrating the identification of relevant literature.

A list of the studies that were excluded, as well as the reason for exclusion, is presented in S2 Appendix.

### Characteristics / overview of included articles

Of the 19 studies, 11 researchers published their work in the last 10 years (2015 onwards). Five studies originated from the USA, three from Australia, and two from UK, while the remaining nine studies came from Saudi-Arabia, Denmark, France, the Netherlands, Malaysia, Turkey, Finland, Norway and South Africa. Fourteen studies employed mixed methodology. Most researchers used thematic analysis or qualitative content analysis. Six studies analyzed litigation claims or complaints made to statutory bodies. Six studies evaluated online reviews of dental services. One study focused specifically on reviews of dental school clinics, while one examined online reviews concerning orthodontists. A detailed summary of the included studies appears in S3 Appendix.

### Quality assessment of included articles

The quality appraisal was done independently by two reviewers (LS and CP) in evaluating the limitations and strengths of the eligible studies and how they differ from each other. We used the JBI-QARI for the appraisal. Most conflicts arose in the interpretation of Questions 6, 7 and 8. We judged the overall quality of most of the studies as fair to good. All the studies stated their methodological approach, rather than their philosophical perspective, which was mostly implied. None of the studies contained a statement locating the researcher culturally or theoretically. Ten of the studies did not address the influence of the researcher on the research. In nine of the studies, no ethics approval or evidence of informed consent from participants could be found. Only five studies contained direct quotations or evidence to suggest that participants and their voices are adequately represented. No studies were excluded from the analysis based on the quality appraisal since they contained findings that were valuable for the analysis. A summary of the methodological quality appraisal of each study appears in Table 1. The supplementary information in the S3 Appendix highlights the diverse contexts and origins of the studies.

**Table 1:** Quality appraisal of included articles.

Authors and year/ Questions	Q1	Q2	Q3	Q4	Q5	Q6	Q7	Q8	Q9	Q10
Alsaeed et al. (2024) [34] Saudi Arabia	Y	Y	Y	Y	Y	N	Y	U	Y	Y
Badenoch-Jones et al. (2015) [35] Australia	Y	Y	Y	Y	Y	N	N	U	N	Y
Bjorndal and Reit (2008) [36] Denmark	Y	Y	Y	Y	Y	N	Y	U	N	Y
Blanchard et al. (2022) [37] England	Y	Y	Y	Y	Y	N	N	U	Y	Y
De Brauwer et al. (2018) [38] France	Y	Y	Y	Y	Y	N	N	U	Y	Y
Eijkman et al. (1984) [39] Netherland	Y	Y	Y	Y	Y	N	Y	U	N	U
Esa et al. (2006) [40] Malaysia	Y	Y	Y	Y	Y	N	N	U	N	Y
Feng et al. (2022) [41] Australia	Y	Y	Y	Y	Y	N	N	Y	Y	Y
Fredericks-Younger et al. (2017) [42] USA	Y	Y	Y	Y	Y	N	Y	U	Y	Y
Gupta and Alraqiq (2022) [43] USA	Y	Y	Y	Y	Y	N	Y	Y	y	Y
Gürdal et al. (2000) [44] Turkey	Y	Y	Y	Y	Y	N	N	U	N	Y
Hiivala et al. (2015) [45] Finland	Y	Y	Y	Y	Y	N	N	U	N	Y
Kroon and Park (2022) [46] Australia	Y	Y	Y	Y	Y	N	Y	Y	Y	Y
Larsen et al. (2022) [47] Norway	Y	Y	Y	Y	Y	N	Y	Y	Y	Y
Meetz et al. (1987) [48] USA	Y	Y	y	y	Y	N	N	U	N	Y
Newsome and Wright (1999) [49] UK	Y	Y	Y	Y	Y	N	Y	Y	N	Y
Postma et al. (2011) [50] South Africa	Y	Y	Y	Y	Y	N	N	U	Y	Y
Sachdeo et al. (2012) [51] USA	Y	Y	y	Y	Y	N	N	U	N	Y
Skrypczak et al. (2020) [52] USA	Y	Y	Y	Y	Y	N	Y	U	U	Y

Note: Questions: (1) Is there congruity between the stated philosophical perspective and the research methodology? (2) Is there congruity between the research methodology and the research question or objectives? (3) Is there congruity between the research methodology and the methods used to collect data? (4) Is there congruity between the research methodology and the representation and analysis of data? (5) Is there congruity between the research methodology and the interpretation of results? (6) Is there a statement locating the researcher culturally or theoretically? (7) is the influence of the researcher on the research, and vice-versa, addressed? (8) are participants, and their voices, adequately represented? (9) Is the research ethical according to current criteria or, for recent studies, and is there evidence of ethical approval by an appropriate body? (10) Do the conclusions drawn in the research report flow from the analysis, or interpretation, of the data? Abbreviations: N, no; U, unclear; Y, yes.

### Synthesis of identified themes and subthemes

We identified four overarching themes describing patients’ perceived negative experiences and complaints when receiving dental care, namely 1) Impeded access to dental care, 2) Unsatisfactory communication, interactions and attitudes, 3) Deficient clinical treatment, and 4) Unethical and unprofessional conduct.

#### Theme 1: Impeded access to dental care.

Impeded access to dental care included three subthemes: (a) poor availability and responsiveness (b) inaccessible premises, and (c) financial concerns.

a) Poor availability and responsiveness

Patients complained when they found it difficult to schedule appointments [40,42–46,51,52]; or when telephones were not answered, when trying to schedule an appointment [34,41,52]. As one patient who tried to access a public clinic stated:

*…give you a number to call…and you never get through… I tried to make an appointment, phone kept ringing out.Tried several times…* [41]

Poor office hours [34,52], and the fact that some dentists do not have emergency facilities [43,44,48], contributed further to patients’ frustration.

Long waiting times were also a major issue for patients receiving dental treatment in both private and public facilities, as well as long waiting lists at public clinics [34,39–41,43,46,48,49,52].

*.. they make appointments for people at the same time.  .. at two o’clock there can be up to eight people waiting and everybody is looking and wondering ‘well I wonder what time her appointment is?’ or ‘he got here after me but he’s being seen before me.’ The last time I went one guy got up and stormed out. He’d watched four people go in although his appointment was at two o’clock. I told the receptionist they shouldn’t do that, it’s not fair.* [49]

*.. the dentistry is way too slow... I don’t understand why if you have a 10:00 appointment and you’re not seen until 12:30 or 1:00.*

*.. 3 h just to get a single tooth pulled...appointment was at 1:30 didn’t get into the back until 2:45 and left at 6 pm!* [43]

*…wait for the whole day to see a dentist…* [41]

*… been on the waiting list for over 3 years to get my wisdom teeth out.* [46]

Patients also expect to be informed if appointments get cancelled or rescheduled, and not adhering to this may lead to complaints [34,43,46,52].

*My appointments were rescheduled, then cancelled at the last possible moment  ...my appointment cancelled as I walked into the waiting room.* [46]

Administrative inefficiency and disorganized systems further contributed to patients’ negative experiences when dealing with dental offices [40,43,44,46,52].

*..they forgot to book the anaesthesiologist Cannot even provide the correct details regarding payments Called me the other day  .. asked me if I wanted to make an appointment, I said not right now. Next day, send me a text reminding me I have an appointment...* [46]

b) Inaccessible premises

Patients also complained about inaccessible premises [34,42–44,46,48,52]; with inconvenient locations [44,48,52], and difficulty with parking [41–43,46,52], mostly leading to their dissatisfaction.

*…parking is a nightmare…* [41]

*…your car will be clamped if you park for more than 2 hours The receptionist advised us there is 2-hour FREE parking  ... cost $65 in parking fine.* [46]

c) Financial concerns

The affordability of dental treatment was also a big concern, and 11 studies reported complaints about the high cost of dental treatment [34,41–46,49–52].

*… charge like wounded bulls* [46]

#### Theme 2: Unsatisfactory communication, interactions and attitudes.

Poor communication was noted in several studies [34,36,39,41,43–49,51,52]. Patients complained that dentists were uncommunicative, not very interested in them and just wanted to get on with the work. As one patient stated:

*.. the sort of person who got you into the surgery, said ‘Hi’, got the drill out and started almost straightaway. I think if you ask them, they will help you but if you don’t bother asking any questions they will just say ‘Good morning’ and get on with it* [49].

Patients were frustrated by the fact that dentists did not communicate or explain procedures or the cost of procedures to them.

*Didn’t explain the procedure until it was completed...... did not even explain anything other than the minor details about the procedure  .. wanted to get straight down to it when we were only there for a consultation.* [46]

When it comes to informing patients about the costs of treatment dentists also tend to be uncommunicative and vague, as patients noted:

*It might be nice to have a basic idea. By the time you have opened your mouth and he says you need this, that and the other and you have no idea what the charges are for a clean-up or a filling or a crown.’ They are not used to being pinned down are they? I wonder how many times people actually ask them ‘What exactly am I paying for?’ I bet they are not used to that. You are in there and you don’t get a chance to have a chat with them do you?’* [49]

Some patients also blamed dentists for using too many technical terms, making it difficult for them to understand.

*A lot of dentists use too many technical terms that the average person on the street doesn’t understand or know anything about. I think they should be taught to use more basic language.* [49]

*I have to ask a lot to get to know what I have to do.* [47]

Dentists were also blamed for not listening to patients and displaying poor bedside manners [43,52]. A lack of empathy, respect and compassion was noted by patients [43,46,47,49,52]; and patients accused dentists of not putting them at ease and not understanding their fear of dentists.

*… not a good experience for a person with dental anxiety.… makes me nervous and insecure …* [47]

*I said to the dentist that I was scared, I was shaking and sweating and he just looked at me and said ‘Don’t be stupid’. He didn’t put me at ease at all! I left him half way through treatment.’* [49]

Patients had the same sentiment regarding the treatment of children and patients with special needs:

*…Had two very bad experiences with my two kids… dentist was very rude and impatient… …they are awful and have no idea how to treat children.* [41]

*.. nervous dentist goer with a mild intellectual disability. Dentist [had no] skills in calming anxious patients.* [46]

Complaints about poor attitude [34,42,46,49,52], and treating patients rudely and condescendingly [41,46,47,50,52], were also listed.

*I perceive him as a rude and uncomfortable person.* [47]

*…dentist was very rude and impatient…* [41]

*I left in tears. Not because of the extraction itself, but because of the arrogant, mean and totally rude comments made to me by the dentist about the condition of my teeth Not empathetic at all and no emotional intelligence...* [46]

Patients suggested that dentists were not always open to communicating with them regarding complaints about poor treatment [46], and as one patient stated:

*… unhappy with the work and when I complained to them, they fobbed me off and said the problem was with me  .* [46]

Complaints about poor attitude and rudeness also extended to staff, especially the reception staff.

*Even his receptionist seemed to be on the same wavelength as him... ‘Don’t be stupid!’* [49]

*.. feel the negative attitude towards you  ... staff member was so rude to the person, they were speaking loudly and rude and everyone could hear them, no respect to someone privacy* [46].

*…rude receptionist… The receptionist girls who served me are very unhelpful.* [41]

#### Theme 3: Deficient clinical treatment.

Deficient clinical treatment included two subthemes: (a) poor quality of dental treatment, and (b) inadequate aftercare and safety.

a) Poor quality of dental treatment

It is pertinent to note that all the included studies contained complaints about the quality of the clinical treatment. Patients complained about the clinical or professional skills of dentists and poor quality of work being delivered [34–52]

*Had to change dentist to get my tooth fixed properly, because she did not get it right after two tries* [47].

*… think twice before you go here because you will more than likely be returning on a weekly basis for them to rectify their work…* [46]

Moreover, patients complained when expectations were not met.

*…results were poor and the yellow colour from my tooth underneath the composite veneer was easily visible*

*… I did a root canal here and my teeth still hurt, and it is darkened so much.* [46]

Patients also complained that restorations did not last, causing them an inconvenience of sometimes having to revisit the dentist more than once to get this fixed.

*I had a Veneer put on to my front tooth, and within 3 weeks it has come off..... filling that had me coming 8 times to the clinic. A filling that was redone 3 TIMES!!!... filling put in and fell out two hours after and can’t get back to fix it* [46].

This was also the case for dental school clinics, with patients expressing their concern that dentists and students practice on their teeth until they break.

*they’re not trying to fix your teeth they practice on it until they break it and you’ll have to extract it after all the trauma, worst experience.* [43]

Treatment that required multiple visits or that took too long to complete, often with limited or no results, also caused dissatisfaction among patients [36,40,43,44,46,52].

*… waisted 3 years of time and payments  ...now left with corrective procedures to fix errors Had orthodontists here clown around with my teeth for over 6 years. One would get them OK until a new amateur would practice on me and mangle my teeth.* [46]

Many studies listed pain as a major reason for complaints [34,36–38,40,41,45–50,52]. Some patients found the procedures traumatic and unbearable, while other patients accused dentists of rough treatment and applying excess force.

*… vivid disturbing memories of the process, including cracking of my teeth and the nurse walking away causing water to pool in my throat and choking me.* [46]

*…barbaric removal of…teeth…* [41]

*…it felt like they were going to break my whole jaw, it was torture* [46].

Patients also complained that the pain often extended post-treatment.

*‘When I had my wisdom teeth out nobody told me that I was going to be in absolute agony and bleeding for days and days. I was devastated.* [49]

*.. spent my days with pain and pain killers until Christmas day.* [46]

Concerns regarding treatment planning (including inappropriate treatment plans, sequence of treatment not followed, and disagreement with treatment plans) were also noted in several studies [34,40,42,46,50,52].

Not meeting patients’ expectations regarding the dental office environment and equipment not being in working condition contributed further to dissatisfaction [37,43,46,47,49].

*… worst experience, no facility, no fully equipped, even small things as x-ray apron …worst dental practice I have been to - wornout equipment and second grade dentists* [46].

*Worn-down waiting room, where should one hang ones coat? […] could also invest in a new operation lamp, as it sometimes worked and sometimes did not work.* [47]

*I don’t like hearing what’s happening in the next surgery – they should soundproof things better.* [49]

b) Inadequate aftercare and safety

Patients also complained that dentists failed to provide adequate aftercare [35,37,43,46].

*After the surgery, [the provider] simply let me walk out of the room right away without giving me any directions about how to prevent possible infections or tell me to make a revisit appointment.* [43]

Not receiving proper aftercare sometimes resulted in severe circumstances, as one patient stated:

*…left two very small but noticeable shards of the tooth in my gum. The shards became infected and the infection spread to my gums* [46].

Patients also noted instances where they experienced over-medicating by providers.

*… after wisdom teeth surgery I was admitted to the hospital at 2am because I had been overdosed with the prescribed pain killers/medication  ... too many antibiotics recommended by them which unnecessary…* [46]

Several studies reported that dentists do not adhere to proper infection control measures [34,42–46,49,50,52], thereby putting patients at risk and sometimes leading to post-op infections [36,37].

*A dentist is where you have minor operations, you think of it as a hospital. A hospital has to be clean and clinical so why not the dentist?* [49]

#### Theme 4: Unethical and unprofessional conduct.

Lack of informed consent or a failure of service providers to warn patients of the risks of treatment was a pertinent reason for complaint and was listed in almost three-quarters of the studies [35–40,42–46,49,50,52]. Several studies also listed complaints that related to unprofessional conduct of service providers. Patients complained that dentists displayed disruptive behavior [43] or even assaulted them [50], commented on or criticized colleagues in front of them [52], and that they did not treat all patients fair and equal [39,40,43]. There were also complaints that dentists relocated without informing patients [50], that they engaged in unethical advertising [50] and in spamming of patients [46].

*Called every day for almost a week  ... harass you months after your check-up.* [46]

Patients also reported that dentists were unfit to practice owing to psychological disturbance or practicing while impaired, either due to health reasons or while under the influence of alcohol [45,52]. Dentists were also accused of being dishonest and lying to patients and that they cannot be trusted [34,45,50,52]. Failure of service providers to accept accountability also contributed to complaints [43,52], as one patient noted:

*some of their surgeons are bad news...Very unethical and willing to hide from their mistakes, even to the point of not telling the patient who operated on them*… [43]

Complaints related to fraud were also reported [37,50] and included claiming for services not rendered, changing of service dates, overservicing of patients or performing unnecessary tests or operations. Poor record keeping and not giving patients access to medical records also led to patient complaints [35,37,45,50].

Negligence also led to complaints and included claims of incorrect treatment, medication errors, treatment errors and wrong-site surgery or wrong tooth extraction [36–38,42,46,50].

*…they pulled the wrong tooth while under anaesthesia numbness hides it.* [46]

Misdiagnosis, wrong diagnosis, failure to treat, failure to perform tests, failure to prescribe antibiotics, failure to take x-rays and failure to treat emergencies were noted by patients [35,37–39,41–43,50]. Negligence extended to cases when service providers failed to recognize complications [37,42], and when they failed to ask for help or refer patients to specialists [35,37,43,50,52].

### Meta-summary

We grouped the findings and calculated the effect size in a meta-summary, as proposed by Sandelowski and Barroso [25] for the purpose of making the findings more accessible to the reader. The meta-summary is presented in Table 2. All four themes were found in 11 of the 19 studies. It is also evident that “deficient clinical treatment” was the most common concern (100%) found in all of the 19 studies, followed by “unethical and unprofessional conduct” (89%) found in 17 of the 19 studies. The high inter-study presence of “deficient clinical treatment” and “unethical and unprofessional conduct” suggests that concerns about clinical competence and integrity are nearly universal across the available qualitative literature.

**Table 2:** Contribution of articles to themes.

Theme	Impeded access to care			Unsatisfactory communication, interactions and attitudes	Deficient clinical treatment		Unethical and unprofessional conduct	Intra-study effect size*
Sub-theme	Poor availability & responsiveness	Inaccessible premises	Financial concerns		Poor quality of treatment	Inadequate aftercare and safety		
Alsaeed et al. (2024) [34]	S	S	S	S	S	S	S	100%
Badenoch-Jones et al. (2016) [35]					S	S	S	50%
Bjorndal & Reit (2008) [36]				S	S	S	S	75%
Blanchard et al. (2022) [37]					S	S	S	50%
De Brauwer et al. (2019) [38]					S		S	50%
Eijkman et al. (1984) [39]	S			S	S		S	100%
Esa et al. (2006) [40]	S				S	S	S	75%
Feng et al. (2022) [41]	P	P	S	P	P		P	100%
Fredericks-Younger et al. (2017) [42]	S	S	S	S	S	S	S	100%
Gupta & Alraqiq (2022) [43]	P	S	S	P	P	P	P	100%
Gürdal et al. (2000) [44]	S	S	S	S	S	S	S	100%
Hiivala et al. (2015) [45]	S		S	S	S	S	S	100%
Kroon & Park (2022) [46]	P	P	P	P	P	P	P	100%
Larsen et al. (2022) [47]				P	P		P	75%
Meetz et al.(1987) [48]	S	S		S	S			75%
Newsome & Wright (2000) [49]	P		P	P	P	P	P	100%
Postma et al. (2011) [50]			S	S	S	S	S	100%
Sachdeo et al. (2012 [51]	S		S	S	S			75%
Skrypczak et al. (2020) [52]	S	S	S	S	S	S	S	100%
Inter-study effect size**	74%			79%	100%		89%	

*Individual study contribution to themes; **Contribution of each article to themes. P: Primary qualitative data available in the form of direct quotations. S: Secondary qualitative data in the form of specified themes.

To examine the appropriateness of treating the included studies as a single higher-order case, the final themes were compared across the major complaint sources represented in the dataset, including online complaints, regulatory complaints and litigation or malpractice-related claims. This comparison examined whether themes were evident across different complaint sources and whether particular meanings appeared to be context specific. The higher-order case was defined by the shared object of inquiry – qualitatively documented patient complaints concerning dental care – rather than by a common complaint mechanism, healthcare setting or methodological approach. The subgroup comparison is presented in Table 3. Theme 3 (deficient clinical treatment) was present across all the subgroups. Theme 1 (impeded access to care) was not found in the litigation subgroup but was present in the regulatory claims subgroup. The litigation subgroup was dominated by complaints related to “deficient clinical treatment” and “unethical and unprofessional conduct” (theme 4). Theme 2 (unsatisfactory communication, interactions and attitudes) was not present in the military subgroup while theme 4 was not present in the insurance subgroup. All four themes were present across online private -, dental schools -, regulatory – and population subgroups. The absence of “impeded access to care” in litigation-based studies likely reflects the legal framing of such complaints rather than a lack of concern about access per se, reinforcing the value of synthesizing across heterogeneous complaint sources.

**Table 3:** Subgroup comparison of theme distribution.

Study	Complaint source	Impeded access to care			Unsatisfactory communication, interactions and attitudes	Deficient clinical treatment		Unethical and unprofessional conduct
		Poor availability & responsiveness	Inaccessible premises	Financial concerns		Poor quality of treatment	Inadequate aftercare and safety	
Alsaeed et al. (2024)	Online Private	x	x	x	x	x	x	x
Kroon & Park (2022)	Online Private	x	x	x	x	x	x	x
Larsen et al. (2022)	Online Private				x	x		x
Skrypczak et al. (2020)	Online Private	x	x	x	x	x	x	x
Feng et al. (2022)	Online Public	x	x	x	x	x	x	x
Gupta & Alraqiq (2022)	Online Dental School	x	x	x	x	x	x	x
Fredericks-Younger et al. (2017)	Dental School	x	x	x	x	x	x	x
Gürdal et al. (2000)	Dental School	x	x	x	x	x	x	x
Sachdeo et al. (2012	Dental School	x		x	x	x		
Badenoch-Jones et al. (2016)	Litigation					x	x	x
Bjorndal & Reit (2008)	Litigation				x	x	x	x
Blanchard et al. (2022)	Litigation					x	x	x
De Brauwer et al. (2019	Litigation					x		x
Hiivala et al. (2015)	Regulatory	x		x	x	x	x	x
Postma et al. (2011)	Regulatory			x	x	x	x	x
Esa et al. (2006)	Military	x				x	x	x
Eijkman et al. (1984)	Population	x			x	x		x
Newsome & Wright (2000)	Population	x		x	x	x	x	x
Meetz et al.(1987)	Insurance	x	x		x	x		

## Discussion

This qualitative meta-synthesis of the literature on patient complaints in dentistry yielded four interrelated domains of concern, namely impeded access to dental care; unsatisfactory communication, interactions, and attitudes; deficient clinical treatment; and unethical and unprofessional conduct. That patients complain about these issues has been documented before in context-specific studies and quantitative complaint analysis. This study integrates these fragmented findings into a coherent conceptual understanding of patient complaints and their dissatisfaction that transcends individual complaint categories and highlights the interconnected nature of patients’ experiences.

Deficient clinical treatment and unethical conduct emerged most prominently in the included studies, but the findings also suggest that, within this corpus, patients’ complaints are often described as extending beyond technical aspects of treatment and as involving relational, organizational and systemic dimensions of dental care. Patient complaints in these studies can thus be interpreted as embedded in the broader conditions under which care is accessed, delivered, communicated and governed. In this sense, adverse clinical outcomes appear in many accounts as only one element in a constellation of concerns that also encompasses how patients are treated as persons and how the system responds to their needs. Within this synthesis, complaints may therefore be understood not simply as reactions to discrete technical failures but as multidimensional and relational phenomena. Across the included studies, patients frequently valued respect, transparency, responsiveness and ethical conduct as integral to their sense of competent care, and they often framed their dissatisfaction in terms of feeling ignored, disrespected, unsafe or misled. The prominence of communication and relational concerns supports the view that patient dissatisfaction in dental care is not adequately captured by technical treatment outcomes alone. Rather than isolated responses to single events, formal complaints, as documented here, can be interpreted as expressions of how patients experience access, interactional quality, clinical safety and professional integrity in combination.

The four domains of complaints appear to converge in pointing to underlying processes in which patients experience threats to trust, patient agency and autonomy, relational security, and the fulfilment of professional and ethical obligations. From this relational-ethical perspective, this convergence suggests that complainants frequently perceived that the fiduciary relationship between patient and practitioner had been compromised. A compromised fiduciary relationship is thus our interpretive inference that coherently captures the patient complaints, whether through impeded access to dental care, unsatisfactory interactions, deficient clinical treatment, or unethical and unprofessional conduct. Considered in this way, impeded access to dental care may be experienced as signaling that patients’ needs and time are not sufficiently valued [53,54]. Communication failures may be experienced as diminishing patients’ sense of agency and the respect they expect from their dentist [55,56]. Deficient treatment and unfavorable conduct may weaken confidence in the dentist’s professional competence [56–58]. Unethical conduct may erode perceptions of the dentist’s integrity and trustworthiness [56,59–61]. Formal complaints, from this perspective, may represent the culmination of gradually deteriorating trust in the therapeutic relationship rather than a response to a single unsatisfactory outcome.

This meta-synthesis yielded furthermore a similarity of complaints across diverse contexts, with studies originating from various countries, differences in healthcare systems, public and private service settings, regulatory environments, and provisions for raising complaints. The sources of complaints also vary, ranging from litigation records, complaints to regulatory bodies, and online reviews. Despite this heterogeneity, similarities of complaints emerged as synthesized in the four domains of complaints. These similarities suggest that access, communication, clinical competence and professional integrity may represent relatively fundamental dimensions of how patients evaluate dental care and construct negative experiences as complaint-worthy. The findings move thus beyond local descriptions of dissatisfaction and provide a higher-order understanding of recurrent concerns that appear to transcend specific organizational and healthcare contexts.

The synthesis further suggests that patient complaints may be understood as signals of broader systemic and relational functioning. Complaints regarding waiting times and difficulties obtaining appointments may indicate failures of organizational responsiveness [62]. Difficulties obtaining follow-up care may indicate weaknesses in continuity of care [63,64]. Communication complaints may reflect perceived deficiencies in person-centered care and informed consent processes [56,65–67]. Complaints about unethical conduct may be experienced as disruptions in professional trustworthiness and accountability [56,58,59,61,68]. Understood in this way, patient complaints represent more than expressions of dissatisfaction with isolated encounters. They provide patient-generated accounts of how dental systems and patient-practitioner relationships function in practice, and where they are perceived to fall short. The findings therefore support approaching complaints not only as events requiring management, but also as important sources of information for quality improvement, organizational learning, and the identification of professional education and capability needs. Efforts to reduce the incidence and impact of complaints may likewise be informed by this relational and systemic understanding. Organizational responsiveness [13,69], communication skills training and development [55,70], management of patient expectations [71–73], strengthened informed consent procedures [74–76], improved continuity of care, and processes that promote professional accountability and transparency [58,63,77–79] may all contribute to reducing complaints and improving patient experiences.

The present meta-synthesis sought to construct an interpretive understanding of how patient complaints have been expressed across the available body of qualitative literature as a given. It does not produce population-level generalizations regarding patient complaints in dentistry for which quantitative studies are suitable. Rather, drawing on a corpus of nineteen studies, the synthesis may be conceptualized as a bounded higher-order case, with individual studies representing embedded subcases that collectively illuminate the phenomenon of patient complaints in dentistry [26]. This bounded higher-order case is thus similar to a qualitative case-study design. It foregrounds not the frequencies of complaints and categories of allegations, but the similarities of meanings that patients express in their negative dental experiences and the conditions under which such experiences become formal complaints [80–82]. In this sense, the study provides an interpretive account of how patient complaints are expressed as narrated and documented across the available qualitative literature.

### Limitations

This meta-synthesis is qualified by substantial heterogeneity across the included studies, including differences in healthcare and regulatory contexts, sources of complaints data, and methodological approaches. The four domains identified should therefore be understood as an interpretive account of patterns across the available literature rather than an exhaustive representation of complaints in any specific setting; their transferability may vary according to contextual and system-level differences. The restriction to English-language publications may also have limited representation of experiences reported in non-English-speaking settings.

Methodological limitations within the primary studies further affect confidence in the synthesis. Variability in researcher reflexivity, ethical reporting, and representation of participant perspectives, together with the inclusion of mixed-method studies, may have constrained the depth and contextual richness of the available qualitative evidence. Consequently, the findings should be interpreted with appropriate caution, as weaknesses and incomplete reporting in the underlying studies may have limited the confidence with which some higher-order interpretations can be supported. The evidence base may also over-represent particularly salient or negative experiences because complaints and published reports are more likely to arise from experiences perceived as distressing, harmful, or ethically concerning

Furthermore, as with any qualitative synthesis, interpretations are necessarily imbued by the perspectives of the researchers. All three authors are researchers at the same Faculty of Health Sciences who lecture in ethics, communication and interpersonal skills, while two of us (LS & CP) are dental professionals with expertise in dental practice management and leadership. Although the derivation of data and the synthesis process have been subject to the reflexive practices as described in the methods section and have been described transparently and in a potentially reproducible way, our disciplinary backgrounds, professional experiences, and interests may have influenced and shaped the lenses through which we have interpreted and synthesized the findings.

### Practical implications and recommendations

The findings have implications across dental education, clinical practice and healthcare policy. Given that “Impeded access to dental care” -particularly financial concerns – was a recurrent domain of complaints, reducing financial barriers requires greater integration of oral health into universal health coverage, improved public funding for essential and specialized dental services, and coverage models that provide greater financial protection, particularly for vulnerable populations [83–85]. Such reforms should address the comparatively high out-of-pocket costs associated with dental care and improve the affordability and accessibility of treatment.

In light of frequent complaints about communication, deficient aftercare and unethical conduct, dental practitioners should strengthen transparency, communication and continuity of care. Treatment costs, including potential additional or follow-up expenses, should be discussed clearly before treatment begins to support informed financial decision-making and reduce perceptions of unexpected or hidden costs. Person-centered communication should incorporate shared decision-making, plain language, active listening, empathy, opportunities for questions and feedback, and appropriate use of visual information [67,86,87]. Dental teams should also be supported in developing emotional intelligence and interpersonal skills, recognizing that patients’ experiences of care are shaped not only by clinical outcomes but also by how they are treated throughout the care process [88]. Clear communication about the nature of proposed procedures, risks, limitations, alternatives and realistic outcomes is particularly important for managing expectations and maintaining trust.

High-quality clinical care should be accompanied by appropriate quality assurance and continuity-of-care mechanisms. Robust clinical protocols, adherence to evidence-based practice, appropriate referral pathways and systematic follow-up can help identify and address complications or unmet patient needs at an early stage. This is particularly important in dentistry, where technically complex and potentially anxiety-provoking procedures may heighten patients’ vulnerability. Importantly, complaints should not be viewed solely as indicators of clinical failure; they may arise from the interaction between a clinical problem and patients’ experiences of communication, respect, and responsiveness [89]. Complaint management should therefore include attention to both the substantive clinical concern and the relational dimensions of care.

Because many of the complaints centered on how patients were treated, rather than only on technical outcomes, undergraduate dental curricula should explicitly integrate communication, professionalism, ethical decision-making, teamwork, leadership and emotional intelligence alongside technical competencies. These skills should be taught longitudinally and reinforced through experiential learning, reflective practice and assessment rather than treated as discrete or supplementary competencies. Mindfulness and other approaches that promote attentiveness and self-awareness may also support reflective and ethically responsive practice [90]. Patient complaints provide a particularly valuable, practice-based educational resource: anonymized complaints can be incorporated into case-based learning, ethics teaching and communication training to enable students to examine how apparently clinical problems may also involve issues of autonomy, respect, accountability, professional integrity and trust [91]. Continuing professional development should similarly reinforce these competencies across the dental team.

Collectively, these recommendations support a shift from viewing complaints primarily as adverse events or indicators of individual professional failure towards understanding them as signals of potential weaknesses across clinical, relational, organizational and structural dimensions of dental care. A coordinated response spanning education, practice and policy may therefore contribute not only to reducing complaints but, more importantly, to strengthening person-centered care, professional accountability, equitable access and public trust in dentistry.

Context-specific qualitative studies could complement this higher-order synthesis by examining how patients experience, interpret and articulate complaints within particular clinical, cultural, organizational and regulatory contexts, adding contextual depth to the patterns identified across the included studies.

## Conclusion

This meta-synthesis contributes an understanding of complaints about dental healthcare by which an interaction of patient experiences concerns impeded access to dental care; unsatisfactory communication, interactions, and attitudes; deficient clinical treatment; and unethical and unprofessional conduct. It suggests the formal complaints may be best understood as indicators of breakdowns in the fiduciary patient-practitioner relationship and, as such, constitute an important source of insight into the relational and systemic conditions necessary for trustworthy, person-centered dental care.

The four domains yielded by this meta-synthesis, from a patient perspective, suggest areas in which aspects of dental health systems may be experienced as functionally deficient. The domains may also point to compromised integrity of patient-practitioner relationships. It may similarly inform directions for organizational or institutional learning and for tailoring education, training and professional capability development that are intended to enhance the quality of dental care and address sources of patient complaints.

## Supporting information

S1 Appendix PRISMA Checklist. (DOCX)

S2 Appendix Summary of excluded articles. (DOCX)

S3 Appendix Summary of articles included in the meta-synthesis. (DOCX)
