# Supplementary material for: Patient complaints in dentistry: A qualitative meta-synthesis
Source: PLoS One. 2026 Nov 12;21(11):e0362563. doi: 10.1371/journal.pone.0362563 (PMC13759093; doi:10.1371/journal.pone.0362563)
Supplement: Supplementary file 1 [file pone.0362563.s001.docx]

S3 APPENDIX

Summary of articles included in the meta-synthesis

| Author (year), Country | Title | Aim/Objectives | Setting | Design / methodology | Key findings |
| --- | --- | --- | --- | --- | --- |
| 1. Alsaeed et al. (2024), Saudi Arabia | Content analysis of online reviews for dental clinics in Saudi Arabia | To examine the content, prevalence and distribution of Google reviews for dental clinics in three major cities in Saudi Arabia | Dental Clinics in 3 major cities in Saudi Arabia: Riyadh, Jeddah and Dammam | Observational study  Mixed-method  (Categorization of negative reviews in domains and sub-domains) | Negative revies classified in 6 domains and then 17 sub-domains:   1. Bad experience  - Bad experience  2. Clinical skills  - Bad outcome (not finished – complications) - Pain/ uncomfortable procedures  3. Interpersonal skills  - Non-hygienic/ reusing single-use items - Poor personality / social skills from dentist - Poor personality/ social skills from staff  4. Professional skills  - Dishonest/ distrust - Long waiting time - Poor communication skills by dentist - Poor communication skills by staff - Inaccessible/ no reminder calls - Scheduling errors or changes  5. Treatment cost  - Too expensive - Extra (hidden) cost/ price changes/ overcharges - Paid for incomplete service / no refunds  6. Office characteristics  - Office cleanliness - Poor office hours - Office design/ poor accessibility |
| 2. Badenoch-Jones et al. (2015), Australia | The Australian litigation landscape – oral and maxillofacial surgery and general dentistry (oral surgery procedures): an analysis of litigation cases | To locate and analyze all publicly available Australian medical negligence cases in the field of oral surgery to identify those issues that prompted litigation or been found to be problematic by the courts | Dental litigation claims in Australia  Judicial cases dealing with medical negligence in field of general dentistry (oral surgery procedures) and oral and maxillofacial surgery | Qualitative  Thematic analysis | 11 cases involved determination whether practitioners failed in their **duty of care**  6 cases found **negligence**  **Damages awarded in 6 cases**  Issues leading to litigation:   - Failure to warn of material risk - Referral - Inappropriate treatment plan or skill - Duty of care (follow up) - Diagnosis - Medical records - Radiographs |
| 3. Bjorndal and Reit (2008), Denmark | Endodontic malpractice claims in Denmark 1995 - 2004 | To study the reasons for and outcome of malpractice claims handled by the regional and national Danish Dental Complaint Boards from 1995 to 2004 | Danish dental complaints board claims from 1995-2004 | Mixed method  Claims classified and assigned to 14 categories.  Endodontic treatment category is further sub-categorized. | Endodontic complaints categorized:   1. Technical complications or incorrect treatment 2. Persistent pain 3. Wrong treatment 4. Prolonged treatment 5. Lack of information 6. Other reasons:   Technical complications:   - Defective root filling - Separated instrument - Root perforation - Medicament related - Other (foreign body, nerve damage)   Incorrect treatment:   - Symptoms of infection - Tooth weakened/fracture/extraction - Treatment related persistent pain - Incorrect treatment (not further detailed). |
| 4. Blanchard et al. (2022), England | Hospital dentistry litigation in England: clinical negligence claims against the NHS 2015-2020. | To determine the incidence and characteristics of hospital dentistry clinical negligence claims in England from 2015 to 2020 | Hospital dentistry (secondary care settings, community, prison dentistry) | Mixed method | Clinical negligence included:  Failure/delay in treatment  Failure to warn/gain informed consent  Inappropriate treatment  Operator error  Tooth injury etc.  Litigation claims by injury included:  Dental damage  Unnecessary pain  Nerve damage  Unnecessary operation etc. |
| 5. De Brauwer et al. (2018), France | Causes for litigation and risk management in facial surgery: a review of 136 cases | To analyze possible causes for litigation in facial surgery in an effort to avoid these claims in future | French litigation claims related to facial surgery | Retrospective descriptive study  Mixed method | Reasons for litigation:  Informed consent  Discrepancy between expected and achieved result  Faulty treatment  Main factors causing litigation:  Unsatisfactory esthetic or functional result  Nerve damage  Medical mistakes and neglect  Failed treatment  Post-operative complications  Exitus letalis  Delayed or incorrect diagnosis |
| 6. Eijkman et al. (1984), Netherland | 873 questions of Dutch dental patients: a challenge to dental health education | To find out whether the Dutch have questions on dentistry, how many they have and exactly what is asked. | Dutch dental patient complaints | Qualitative  Open ended questionnaires sent to 2000 Dutch dental patients, 640 send back with 398 providing answer to request – these answers (751 questions and 122 complaints) were coded | Most frequent complaints found in subcategories 18(Communication) and 15(Treatment)  Subcategory 18 dissatisfaction about:   - difference in treatment possibilities offered to Sickness fund patients and to private patients. - long waiting time - Limited time dentists spend on their patients. - poor information given by dentists about condition of mouth and on nature of treatment.   Subcategory 15 discontent with:   - So much has to be treated all over again when changing dentist despite regular visits to previous dentist. - Alleged incompetence of dentist. - Why dentists fail to do anything about certain deficiencies despite patient’s request. - Dentists tend to recommend denture too readily, despite patient’s reluctance |
| 7. Esa et al. (2006), Malaysia | Satisfaction with the oral health services. A qualitative study among non-commissioned officers in the Malaysian armed forces | To assess patients’ satisfaction and dissatisfaction with the dental services of the MAF | First Brigade Rasah Camp, Seremban, Malaysia | Qualitative:  Nominal group technique | Total of 15 factors/items caused dissatisfaction – sorted in 3 major categories: administrative efficiency, patient-personnel interaction, technical competency  Admin efficiency: non-availability, long waiting time, sequence of treatment not followed (preference to rank), accessibility  Patient-personnel interaction: uncooperative staff, not courteous, friendly approachable etc.  Technical competence: quality of treatment, rough treatment, hasty manner, treatment not completed in one sitting, fractured fillings, painful extraction, long healing time after extraction, inadequate medicine, no explanations before and after treatment |
| 8. Feng et al. (2022), Australia | Perceptions of service quality in Victorian public dental clinics using Google patient reviews | 1. Quantitatively and qualitatively examine public dental patients’ positive or negative reviews on Google 2. Determine if clinics from different levels of remoteness had different patient reviews | Australian public dental clinics | Mixed method study gathered Google reviews published pre-March 2021  Reviews coded and subthemes and major themes identified | Qualitative:  Major themes of dissatisfaction: treatment, staff, communication and professionalism, patient factors, non-clinical reasons.  Extractions and restorations:  Pain during procedure and failing treatment  Dissatisfied with receptionist – dislike mannerism and number of phone calls being answered  Dissatisfied with how children were treated – concern about dental caries being undetected, dislike mannerism of staff  Dissatisfaction with waiting lists and waiting room  Dissatisfied with parking |
| 9. Fredericks-Younger et al. (2017), USA | Developing a relevant taxonomy to assess dental school clinic patient complaints | To categorize and analyze the most frequent patient complaints at the RSDM clinics in an effort to identify areas in need of improvement. | Rutgers School of Dental Medicine, USA | Mixed  Retrospective analysis  Used coding taxonomy to categorize complaints | Developed new complaint taxonomy   1. **Clinical**  - Patient journey - Treatment coordination - Treatment delays - Treatment plan - Perception - Expectations - Patient responsibility - Preexisting condition - Recognition of normal complications - Quality of care - Defective treatment - Infection control - Treatment error - Relationships - General dissatisfaction with student/resident/faculty - Informed consent - Patient rights  2. **Operations**  - Finance - Cost of treatment - Fee schedule - Policy - Institution - Access - Bureaucracy - Dissatisfaction with staff - Environment |
| 10. Gupta and Alraqiq (2022), USA | Patient experiences at dental school clinics: A comparative analysis of online reviews | To characterize patient-generated reviews posted on the yelp and Google review sites and assess any associations between dental schools’ ratings and their respective characteristics | Online reviews of dental school clinics in USA | Mixed method  Qualitative: code and organized in themes and sub-themes. | Majority of reviews were negative, particularly concerning pre-care and post-care experiences. Common negative themes included issues with staff helpfulness, billing and payment processes, and clinic operations |
| 11. Gürdal et al. (2000), Turkey | Factors of patient satisfaction / dissatisfaction in a dental faculty outpatient clinic in Turkey | Investigate satisfaction with dental care among patients of a dental faculty outpatient clinic of a major university in Izmir, Turkey | Outpatient Dental Clinic of Ege University in Izmir, Turkey | Mixed method  Self-administered questionnaire and focus groups  Content analysis and coding | Components of dissatisfaction:  Disorganized service system  High cost  Long treatment span  Long duration till appointment date  Slowness of radiological examination procedure  No information regarding treatment to be received  Poor previous treatment experiences  No emergency facilities  Insufficient scientific ability of dental personnel  Unsatisfactory sterility/cleanliness  Uncomfortable environment  Inappropriate location |
| 12. Hiivala et al. (2015), Finland | Can patients detect hazardous dental practice? A patient complaint study | To describe patient/family-generated dental complaints during a 12-year period  To provide insight into reasons why patients/families lodge complaints or contact healthcare supervisory authorities  (rest of aims p 275) | Finland dental care | Mixed method  Content analysis of complaints | 1. Patient safety perceived as compromised:  - Patient safety incident or medical error - Access to care delayed, causing harm - Hygiene/infection control inadequate - Practice impaired - Other  2. Other dissatisfaction  - Unprofessional conduct or behavior - Communication (interaction quality, information inadequate, concent lacking, errors - Payments (e.g. overcharge) - Patient records or medical certificates - Other   Post-treatment symptoms: pain or eating problems  Psychological aspects: loss of trust or other emotional distress |
| 13. Kroon and Park (2022), Australia | Negative reviews online: an exploratory analysis of patient complaints about dental services in Western Australia | To identify common themes within dental practice complaints by analyzing one- and two-star Google reviews received by dental practices within Perth metropolitan region.  Study aimed to examine common complaints by patients through online reviews regarding public and private WA dental practices and further identified specific areas of concern within these reviews. | Perth Metropolitan dental clinics  Public and private WA dental practices. | Mixed method  Qualitative component: coding of data into themes  Retrospective cross-sectional analysis | 5 themes:   - Treatment issues - Staffing - Communication and professionalism - Finance - Premise   38 subthemes  Treatment issues primary concern  Treatment dissatisfaction also high within subthemes |
| 14. Larsen et al. (2022) Norway | Challenging encounters in clinical dentistry: a qualitative study investigating online reviews of patient satisfaction with Norwegian dentists | To map key themes that characterise challenging and positive encounters in dental practice using online reviews of patient satisfaction with Norwegian  dentists. | Dental practice online reviews in Norway published on Legelisten.no | Qualitative  Thematic analysis | The thematic analysis revealed five central themes:   1. **Interpersonal Factors**: Poor communication and perceived lack of empathy were associated with challenging encounters. (uncommunicative, rude, seemed uninterested in wellbeing) 2. **Patient Factors**: Negative past experiences or heightened anxiety could predispose patients to view encounters as challenging. 3. **Dentist Factors**: Perceived rudeness or haste contributed to negative reviews. (stressed or in a hurry while treating patient, traits like aggression, disrespect, introversion) 4. **Situational Factors**: Factors like long waiting times or a chaotic clinic atmosphere were associated with challenging encounters. 5. **Consequences**: Complications or unmet expectations resulted in dissatisfaction. (severe pain after and during treatment, worsening of dental health, more anxiety related to dental treatment).   The study concluded that challenging encounters often arise when there is a mismatch between dentists' communication styles and patients' expectations or preferences. |
| 15. Meetz et al. (1987), USA | A content analysis of dental capitation plan grievances | To examine the aspects of patient dissatisfaction with delivery of health services in a capitated plan. | Dental capitation plan patients in California | Mixed method  Qualitative involved content analysis and coding | Grievance categories included:  Plan coverage, Patient-provider communication, Access to care, Provider technical competence, Plan administration and provider facilities  Access to care largest concern   - Wait for appointment - wait in office - unavailability for emergencies - distance from office   Provider technical competence second   - Provider competence / request for second opinion - Request for specialist opinion - Pain/discomfort suffered by patient   Patient-provider communication:   - Language barrier - Provider-patient communication - Staff-patient communication |
| 16. Newsome and Wright (1999), UK | Qualitative techniques to investigate how patients evaluate dentists: a pilot study | To explore the following issues:   - What do people expect from their dentist - Upon which aspects of dentists and dental practice do patients hold expectations and form perceptions? - How do expectations and perceptions interact in the evaluation process? - How, and under what circumstances do patients apportion blame? | English-speaking adults (over 18) in England town of Heckmondwike who attended dental treatment in previous 12 months | Qualitative  Focus group interviews  Code mapping of data | Expectations least likely to be met in area of dentist-patient communication  If patient’s problem, goal or request falls within his /her perception of dentist’s duty and patient perceives the dentist to be culpable for any negative experience then that patient is likely to be dissatisfied. Perceived “duty” may be regarded as a form of expectation that embodies an individual’s perception of what his or her rights are in relation to the service and what the service’s obligations are to him/her.  Perceptions organized in:   - Dentist’s attitude and behavior - Dentist’s approach to pain control and reduction of anxiety - Dentist’s ability to solve their dental problems - Staff attitudes and behavior - Convenience factors - Physical cues |
| 17. Postma et al. (2011), South Africa | An analysis of complaints against oral health professionals charged with misconduct at the HPCSA: 2004-2009 | To investigate the nature, frequency and outcome of the complaints laid against oral health practitioners charged with misconduct by the HPCSA | Complaints against oral health professionals in South Africa | Mixed method  Nature and outcome of complaints and the penalties were quantified, and the detailed nature of the complaints was qualitatively reported. | Complaint categories for dentists:  Clinically related  Fraud  Poor record keeping  Rude language  Unethical advertising  Assault  Employing an unregistered person  Poor infection control  Practice while suspended  Clinical related grievances and fraud accounted for most common complaints. |
| 18. Sacdeo et al. (2012), USA | An analysis of patient grievances in a dental school clinical environment. | To collect and  categorize all the complaints received from patients seeking treatment at Tufts University School of  Dental Medicine (TUSDM) from 2005 to 2008 | TUSDM clinics,  which serve the greater Boston area in Massachusetts,  with additional patients mainly from Boston and New England | Mixed method  Coding and categorization | The complaints were categorized into five main areas:   1. **Appointment Issues**: Difficulties related to scheduling and maintaining appointments. 2. **Communication**: Problems arising from interactions between patients and dental care providers. 3. **Financial Concerns**: Disputes or misunderstandings regarding treatment costs. 4. **Quality of Care**: Patient dissatisfaction with the standard of dental care received. 5. **Other**: Miscellaneous complaints not fitting into the above categories.   The study found that the majority of complaints pertained to appointment scheduling, followed by communication issues, financial concerns, and quality of care. |
| 19. Skrypczak et al. (2020), USA | Negative online reviews of orthodontists: content analysis of complaints posted by dissatisfied patients | Analyze online reviews of orthodontists with an emphasis on the types and frequency of complaints expressed in negative reviews | Orthodontic patient complaints in USA | Mixed method  Content analysis | Complaint categories:  Quality of care  Interpersonal interactions  Money / financial issues  Time  Communication  Unprofessional behavior  Office characteristics |
