# Supplementary material for: Patient complaints in dentistry: A qualitative meta-synthesis
Source: PLoS One. 2026 Nov 12;21(11):e0362563. doi: 10.1371/journal.pone.0362563 (PMC13759093; doi:10.1371/journal.pone.0362563)
Supplement: Supplementary file 2 [file pone.0362563.s002.docx]

S2 APPENDIX

Summary of excluded articles

| Author (year), Country | Title | Exclusion reason |
| --- | --- | --- |
| Abomalik et al. (2022), Saudi Arabia | A retrospective assessment of the dental malpractice cases filed in Riyadh from 2009-2015. | Unsuitable study design, quantitative study |
| Alsaeed et al. (2022), Saudi Arabia | Dental malpractice lawsuit cases in Saudi Arabia: A national study. | Unsuitable study design, quantitative study |
| Eijkman et al. (1997), The Netherlands | Defensive dental behavior: illusion or reality? | Outside the inclusion criteria, not about patient complaints |
| Felgner et al. (2022), Germany | Reasons for (not) choosing dental treatment – A qualitative study based on patients’ perspective. | Outside the inclusion criteria, not primarily about patient complaints |
| Hopcraft & Sanduja (2006), Australia | An analysis of complaints against Victorian dental care providers 2000-2004 | Unsuitable study design, quantitative study |
| Levey et al. (2015), Norway | Patients give meaning to changes in health complaints before, during and after the replacement of amalgam restorations. | Outside inclusion criteria, not about patient complaints, not a primary study – a qualitative summary |
| Nogueira et al. (2019), Brazil | Perceptions and experiences of patients following treatment with single-implant mandibular overdentures: A qualitative study. | Outside the inclusion criteria, not about patient complaints |
| Schwarz (1988), Denmark | Patient complaints of dental malpractice in Denmark 1983-86. | Unsuitable study design, quantitative study. |
| Thomas et al. (2018), Australia | Complaints about dental practitioners: an analysis of 6 years of complaints about dentists, dental prosthetists, oral health therapists and dental hygienists in Australia | Unsuitable study design, quantitative study |
| Wang et al. (2024), New Zealand | Risk identification and prediction of complaints and misconduct against health practitioners: a scoping review | Wrong population, Unsuitable study design / not a primary study. |
