# Supplementary material for: Patient complaints in dentistry: A qualitative meta-synthesis
Source: PLoS One. 2026 Nov 12;21(11):e0362563. doi: 10.1371/journal.pone.0362563 (PMC13759093; doi:10.1371/journal.pone.0362563)
Supplement: Supplementary file 4 [file pone.0362563.s004.docx]

**Review on: Patient complaints in dentistry: A qualitative meta-synthesis**

**Major concerns**

1. The central conclusion that patient complaints represent "breakdowns in the fiduciary patient-practitioner relationship" appears to exceed the evidence presented in the Results section. The authors should demonstrate more explicitly how the primary data support this interpretation and clarify whether this framework emerged from the data or was informed by pre-existing ethical theory.

The author should also consider and discuss alternative explanatory frameworks for patient complaints such as patient expectations, service quality, healthcare accessibility, and patient safety.

2. A major methodological concern is the inclusion of highly diverse sources of complaints, including litigation claims, regulatory complaints, online reviews, dental school complaints, and public dental service complaints.

These represent fundamentally different forms of patient feedback with different thresholds, motivations, and contexts. The authors should provide stronger justification for synthesizing these sources as a single higher-order case or provide subgroup comparisons demonstrating thematic consistency across complaint types.

3. Fourteen of the nineteen included studies were mixed-methods studies.

The manuscript insufficiently explains:

How qualitative findings were isolated from quantitative findings.

How qualitative data were extracted.

Whether quantitative complaint categories influenced coding and theme development.

Greater transparency regarding data extraction procedures would strengthen methodological rigor and reproducibility.

4. The quality appraisal demonstrates important weaknesses among included studies.

The authors report that:

No study adequately addressed researcher positioning.

Many studies lacked reflexivity.

Several studies lacked ethical reporting.

Only a minority presented direct participant quotations.

Despite these limitations, all studies were retained in the synthesis.

The manuscript would benefit from:

The author should state the implications of methodological weaknesses for confidence in the findings.

5. The stated objective is to understand the meaning patients express through complaints. However, the quality assessment indicates that only a small number of studies contained substantial participant quotations.

The authors should discuss how this limitation may affect the depth of interpretive claims and the extent to which it reflects patient voices versus author interpretations from primary studies.

6. The description of the thematic synthesis process remains relatively broad.

The manuscript would benefit from additional detail regarding code development, theme generation, management of disagreements between reviewers, and decisions used to prioritize latent versus semantic themes.

This includes examples of coding progression from raw data to final themes that may increase transparency and trustworthiness.

7. There appears to be conceptual overlap between deficient clinical treatment and unethical and unprofessional conduct.

Examples such as informed consent failures, inadequate follow-up care, negligence, and treatment planning concerns could plausibly fit within either category.

The authors should clarify how coding boundaries were established and maintained during analysis.

8. The authors indicate that searches were completed in September 2024. However, several references cited in the Discussion are from 2025 and 2026.

The authors should specify: Whether the searches were updated, whether citation searching continued after database searching, and the final date on which literature was screened.

This is important for transparency and reproducibility.

9. Several statements in the Discussion appear stronger than warranted by the results.

Examples include assertions that patients perceive that their needs are unimportant, complaints represent the culmination of deteriorating trust, and complaints reflect impaired fiduciary relationships.

These interpretations are plausible but should either be more cautiously phrased or more explicitly connected to supporting evidence from the included studies.

10. Reporting of Review Processes

To strengthen methodological transparency, the authors should consider reporting the following:

Reviewer agreement statistics, number of disagreements during title/abstract and full-text screening, procedures used to resolve disagreements, and additional details regarding audit trails and reflexive documentation.

Such information would improve confidence in the review process.

11. Several minor issues should be corrected: Some quotations are excessively long and could be condensed. There are occasional grammatical inconsistencies and awkward wording.

The phrase "the synthesis so derived" could be revised for clarity.

Table 2 may benefit from clearer explanation or simplification.
