# Supplementary material for: Patient complaints in dentistry: A qualitative meta-synthesis
Source: PLoS One. 2026 Nov 12;21(11):e0362563. doi: 10.1371/journal.pone.0362563 (PMC13759093; doi:10.1371/journal.pone.0362563)
Supplement: Supplementary file 5 [file pone.0362563.s006.docx]

Lorraine Snyman
Department of Dental Management Sciences
Faculty of Health Sciences, University of Pretoria

lorraine.syman@up.ac.za

5 October 2026

RE: Response to reviewer comments

Dear Professor Allam

We appreciate the comments provided in consideration of our article. We have extensively reworked our paper. The following table highlights the responses to the individual reviewer comments.

| **Editor comments** | **Response** |
| --- | --- |
| We note that the most recent literature search was completed over a year before the manuscript was submitted to the journal. Systematic and scoping reviews should provide an up-to-date overview of the research topic covered. Therefore, we request that you please update your searches as part of your revision to ensure that the most up-to-date literature is included in your review, or provide scientific justification for not doing so. | We have updated our database search on 26 August 2026 and adjusted the relevant sections in the methodology and results sections accordingly. The PRISMA flow diagram was adjusted accordingly. No new studies were identified for inclusion in this synthesis. The updated search had no impact on the synthesized results or discussion. |
| **Reviewer #1 comments** | **Response** |
| 1. Figure 1: The resolution of Figure 1 (PRISMA flow diagram) is low. Please provide a higher-resolution version to ensure optimal readability in the published version. | We have updated the PRISMA flow diagram in a higher-resolution version to ensure optimal readability. |
| 2. Keywords (Line 47): Please ensure all listed keywords strictly align with standard Medical Subject Headings (MeSH) terms and consider removing overly generic terms. | We have changed our keywords to align with standard medical subject headings and removed overly generic terms. Key words were changed from “Patient complaints, oral health, dentistry, dental education, ethics” to “dentistry, qualitative research, patient complaints, meta-synthesis” |
| 3. Introduction: While the aim of the study is clearly stated, the manuscript would benefit from an explicit and concise definition of the knowledge gap. Additionally, strengthening the theoretical/conceptual framework in the introduction will better highlight the novel contribution of this synthesis. | We have now added a paragraph to the Introduction to explicitly state the knowledge gap and highlight the novel contribution of this synthesis. We also highlighted the interpretive nature of this synthesis. |
| 4. Methods - Search Update (Line 143): The initial literature search was conducted in September 2024. Please consider updating the search to cover any recent literature published since then, if applicable. | We have updated our database search and adjusted the relevant sections in the Methodology and Results sections accordingly. The PRISMA flow diagram was adjusted accordingly. No new studies were identified for inclusion in this synthesis. |
| 5. Results - Presentation of Direct Quotes: The Results section relies heavily on extensive direct quotes throughout the narrative text. To improve readability and structural flow, consider condensing these quotes within the main text and transferring extended quotes to a supplementary table or appendix. | We have condensed some of the quotations and removed redundancies in the quotes without compromising the meaning but retaining the qualitative evidence. |
| 6. Discussion - Limitations Section (Lines 578–624): The Limitations section is currently overly lengthy and somewhat defensive in tone regarding design choices. Please condense this section to focus more concisely on the key methodological and contextual limitations without over-justifying standard qualitative review constraints. | We have condensed the limitations section in a less defensive tone, focusing on the key methodological and contextual limitations. We also added a statement regarding the implications of the methodological weaknesses of the included studies for confidence in our findings. |
| 7. Conclusion (Lines 626–639): The conclusion effectively synthesizes the main domains; however, it would be considerably strengthened by providing specific, actionable recommendations and practical implications for dental education, clinical practice, and healthcare policy. | We have now added a section “Practical implications and recommendations” to the manuscript and agree that it may considerably strengthen the manuscript and conclusion. |
| **Reviewer #2 comments** | **Response** |
| 1. The central conclusion that patient complaints represent "breakdowns in the fiduciary patient-practitioner relationship" appears to exceed the evidence presented in the Results section. The authors should demonstrate more explicitly how the primary data support this interpretation and clarify whether this framework emerged from the data or was informed by pre-existing ethical theory. The author should also consider and discuss alternative explanatory frameworks for patient complaints such as patient expectations, service quality, healthcare accessibility, and patient safety. | We have rephrased the Introduction to highlight the interpretive nature through a relational-ethical lens. In the Discussion section, we qualify this interpretative inference accordingly and describe the coherence this inference provides in explaining the relevant patient complaints. |
| 2. A major methodological concern is the inclusion of highly diverse sources of complaints, including litigation claims, regulatory complaints, online reviews, dental school complaints, and public dental service complaints. These represent fundamentally different forms of patient feedback with different thresholds, motivations, and contexts. The authors should provide stronger justification for synthesizing these sources as a single higher-order case or provide subgroup comparisons demonstrating thematic consistency across complaint types. | We have now done a subgroup comparison to highlight consistency across subgroups and added the table to the Result section. |
| 3. Fourteen of the nineteen included studies were mixed-methods studies. The manuscript insufficiently explains: How qualitative findings were isolated from quantitative findings. How qualitative data were extracted. Whether quantitative complaint categories influenced coding and theme development. Greater transparency regarding data extraction procedures would strengthen methodological rigor and reproducibility. | We have now revised the Methods section to describe the data extraction and coding boundaries in a more explicit and transparent way |
| 4. The quality appraisal demonstrates important weaknesses among included studies. The authors report that: No study adequately addressed researcher positioning. Many studies lacked reflexivity. Several studies lacked ethical reporting. Only a minority presented direct participant quotations. Despite these limitations, all studies were retained in the synthesis. The manuscript would benefit from: The author should state the implications of methodological weaknesses for confidence in the findings. | This refers partly to comment 6 of Reviewer#1.  We have added a statement in the limitations about the implications of methodological weaknesses of the included studies for confidence in our findings |
| 5. The stated objective is to understand the meaning patients express through complaints. However, the quality assessment indicates that only a small number of studies contained substantial participant quotations. The authors should discuss how this limitation may affect the depth of interpretive claims and the extent to which it reflects patient voices versus author interpretations from primary studies. | This was stated in the methods as well as the limitations section. We have also qualified the interpretive inference in the introduction and discussion section. To improve transparency in the results we have indicated in table 2 the origin of the data as “primary” or “secondary” in relation to their theme contribution. |
| 6. The description of the thematic synthesis process remains relatively broad. The manuscript would benefit from additional detail regarding code development, theme generation, management of disagreements between reviewers, and decisions used to prioritize latent versus semantic themes. This includes examples of coding progression from raw data to final themes that may increase transparency and trustworthiness. | We have revised the Methods section analytic process and have described code progression, theme generation and management of disagreements in a more transparent way. |
| 7. There appears to be conceptual overlap between deficient clinical treatment and unethical and unprofessional conduct. Examples such as informed consent failures, inadequate follow-up care, negligence, and treatment planning concerns could plausibly fit within either category. The authors should clarify how coding boundaries were established and maintained during analysis. | We have revised the Methods section to make the analytic procedure more explicit, and we have added examples illustrating how potentially overlapping material was considered during the analytic process. We also describe now that, by the nature of complaints being experiential, imbrication may be expected rather than being categorically discrete; their boundaries represent analytic distinctions developed to capture the emphasis and predominant meaning of patients’ complaints while accepting the interconnectedness of the experiences. |
| 8. The authors indicate that searches were completed in September 2024. However, several references cited in the Discussion are from 2025 and 2026. The authors should specify: Whether the searches were updated, whether citation searching continued after database searching, and the final date on which literature was screened. This is important for transparency and reproducibility. | This also refers to comment 4 of Reviewer#1  We have updated our database search and adjusted the relevant sections in the Methods and Results accordingly to display this. The PRISMA flow diagram was also adjusted accordingly. No new studies were identified for inclusion in the synthesis |
| 9. Several statements in the Discussion appear stronger than warranted by the results. Examples include assertions that patients perceive that their needs are unimportant, complaints represent the culmination of deteriorating trust, and complaints reflect impaired fiduciary relationships. These interpretations are plausible but should either be more cautiously phrased or more explicitly connected to supporting evidence from the included studies. | We have now rephrased the interpretations more cautiously. See also our response to comment no 1. |
| 10. Reporting of Review Processes To strengthen methodological transparency, the authors should consider reporting the following: Reviewer agreement statistics, number of disagreements during title/abstract and full-text screening, procedures used to resolve disagreements, and additional details regarding audit trails and reflexive documentation. Such information would improve confidence in the review process. | We have now included a phrase to inform readers that an audit trial of the review process was kept in Covidence, and we have included the reviewer agreement statistics. |
| 11. Several minor issues should be corrected: Some quotations are excessively long and could be condensed. There are occasional grammatical inconsistencies and awkward wording. The phrase "the synthesis so derived" could be revised for clarity. Table 2 may benefit from clearer explanation or simplification. | (Also refer to Comment 5 of Reviewer#1.)  We have condensed some of the quotations and removed redundancies in the quotes without compromising the meaning, but retaining the qualitative evidence  The phrase “the synthesis so derived” has been rephrased to “the resulting synthesis”  Table 2 could not be simplified without changing the meaning of the results; additional information was however, added to provide a better understanding and transparency. |

Thank you for considering this manuscript.

Sincerely,

Lorraine Snyman
