# Supplementary material for: Patient complaints in dentistry: A qualitative meta-synthesis
Source: PLoS One. 2026 Nov 12;21(11):e0362563. doi: 10.1371/journal.pone.0362563 (PMC13759093; doi:10.1371/journal.pone.0362563)
Supplement: Peer review history

# Peer review: Decision Letter 0

**DOI:** 10.1371/journal.pone.0362563.r001

**Authors:** Eman Allam

25 Aug 2026

Dear Dr. Snyman,

Thank you for submitting your manuscript to PLOS One. After careful consideration, we feel that it has merit but does not fully meet PLOS One’s publication criteria as it currently stands. Therefore, we invite you to submit a revised version of the manuscript that addresses the points raised during the review process.

Please submit your revised manuscript by Oct 24 2026 11:59PM. If you will need more time than this to complete your revisions, please reply to this message or contact the journal office at plosone@plos.org. When you're ready to submit your revision, log on to https://www.editorialmanager.com/pone/ and select the 'Submissions Needing Revision' folder to locate your manuscript file.

- A letter that responds to each point raised by the academic editor and reviewer(s). You should upload this letter as a separate file labeled 'Response to Reviewers'.
- A marked-up copy of your manuscript that highlights changes made to the original version. You should upload this as a separate file labeled 'Revised Manuscript with Track Changes'.
- An unmarked version of your revised paper without tracked changes. You should upload this as a separate file labeled 'Manuscript'.

If you would like to make changes to your financial disclosure, please include your updated statement in your cover letter. Guidelines for resubmitting your figure files are available below the reviewer comments at the end of this letter.

If applicable, we recommend that you deposit your laboratory protocols in protocols.io to enhance the reproducibility of your results. Protocols.io assigns your protocol its own identifier (DOI) so that it can be cited independently in the future. For instructions see: https://journals.plos.org/plosone/s/submission-guidelines#loc-laboratory-protocols. Additionally, PLOS One offers an option for publishing peer-reviewed Lab Protocol articles, which describe protocols hosted on protocols.io. Read more information on sharing protocols at https://plos.org/protocols?utm_medium=editorial-email&utm_source=authorletters&utm_campaign=protocols.

As the corresponding author, your ORCID iD is verified in the submission system and will appear in the published article. PLOS supports the use of ORCID, and we encourage all coauthors to register for an ORCID iD and use it as well. Please encourage your coauthors to verify their ORCID iD within the submission system before final acceptance, as unverified ORCID iDs will not appear in the published article. *Only*  the individual author can complete the verification step; PLOS staff *cannot*  verify ORCID iDs on behalf of authors.

We look forward to receiving your revised manuscript.

Kind regards,

Eman Allam, PhD

Academic Editor

PLOS One

Journal Requirements:

When submitting your revision, we need you to address these additional requirements.

1. Please ensure that your manuscript meets PLOS ONE's style requirements, including those for file naming. The PLOS ONE style templates can be found at

https://journals.plos.org/plosone/s/file?id=wjVg/PLOSOne_formatting_sample_main_body.pdf and

https://journals.plos.org/plosone/s/file?id=ba62/PLOSOne_formatting_sample_title_authors_affiliations.pdf

2. We note that the most recent literature search was completed over a year before the manuscript was submitted to the journal. Systematic and scoping reviews should provide an up-to-date overview of the research topic covered. Therefore, we request that you please update your searches as part of your revision to ensure that the most up-to-date literature is included in your review, or provide scientific justification for not doing so. Please ensure you mention this in your response to reviewers, and discuss whether this has any impact on the Results and Conclusions of the study. If you are submitting a protocol, it is only necessary to state whether the searches will be updated prior to the publication of the results.

3. If the reviewer comments include a recommendation to cite specific previously published works, please review and evaluate these publications to determine whether they are relevant and should be cited. There is no requirement to cite these works unless the editor has indicated otherwise.

[Note: HTML markup is below. Please do not edit.]

Reviewers' comments:

Reviewer's Responses to Questions

**Comments to the Author**

1. Is the manuscript technically sound, and do the data support the conclusions?

Reviewer #1: Yes

Reviewer #2: Partly

**********

2. Has the statistical analysis been performed appropriately and rigorously?

Reviewer #1: N/A

Reviewer #2: Yes

**********

3. Have the authors made all data underlying the findings in their manuscript fully available?

The PLOS Data policy

Reviewer #1: Yes

Reviewer #2: Yes

**********

4. Is the manuscript presented in an intelligible fashion and written in standard English?

Reviewer #1: Yes

Reviewer #2: Yes

**********

Reviewer #1: Overall, the manuscript addresses a critical and relevant topic in dental care and healthcare management. The review is well-structured, methodologically sound, and provides valuable synthesized insights into patient experiences and complaints in dentistry. The integration of Sandelowski and Barroso's guidelines alongside Hoon’s conceptual framework is well-articulated.

To further enhance the clarity, impact, and presentation of the manuscript prior to publication, I offer the following comments for the authors' consideration:

1. Figure 1:

The resolution of Figure 1 (PRISMA flow diagram) is low. Please provide a higher-resolution version to ensure optimal readability in the published version.

2. Keywords (Line 47):

Please ensure all listed keywords strictly align with standard Medical Subject Headings (MeSH) terms and consider removing overly generic terms.

3. Introduction:

While the aim of the study is clearly stated, the manuscript would benefit from an explicit and concise definition of the knowledge gap. Additionally, strengthening the theoretical/conceptual framework in the introduction will better highlight the novel contribution of this synthesis.

4. Methods - Search Update (Line 143):

The initial literature search was conducted in September 2024. Please consider updating the search to cover any recent literature published since then, if applicable.

5. Results - Presentation of Direct Quotes:

The Results section relies heavily on extensive direct quotes throughout the narrative text. To improve readability and structural flow, consider condensing these quotes within the main text and transferring extended quotes to a supplementary table or appendix.

6. Discussion - Limitations Section (Lines 578–624):

The Limitations section is currently overly lengthy and somewhat defensive in tone regarding design choices. Please condense this section to focus more concisely on the key methodological and contextual limitations without over-justifying standard qualitative review constraints.

7. Conclusion (Lines 626–639):

The conclusion effectively synthesizes the main domains; however, it would be considerably strengthened by providing specific, actionable recommendations and practical implications for dental education, clinical practice, and healthcare policy.

Reviewer #2: Review on: Patient complaints in dentistry: A qualitative meta-synthesis

Major concerns

1. The central conclusion that patient complaints represent "breakdowns in the fiduciary patient-practitioner relationship" appears to exceed the evidence presented in the Results section. The authors should demonstrate more explicitly how the primary data support this interpretation and clarify whether this framework emerged from the data or was informed by pre-existing ethical theory.

The author should also consider and discuss alternative explanatory frameworks for patient complaints such as patient expectations, service quality, healthcare accessibility, and patient safety.

2. A major methodological concern is the inclusion of highly diverse sources of complaints, including litigation claims, regulatory complaints, online reviews, dental school complaints, and public dental service complaints.

These represent fundamentally different forms of patient feedback with different thresholds, motivations, and contexts. The authors should provide stronger justification for synthesizing these sources as a single higher-order case or provide subgroup comparisons demonstrating thematic consistency across complaint types.

3. Fourteen of the nineteen included studies were mixed-methods studies.

The manuscript insufficiently explains:

How qualitative findings were isolated from quantitative findings.

How qualitative data were extracted.

Whether quantitative complaint categories influenced coding and theme development.

Greater transparency regarding data extraction procedures would strengthen methodological rigor and reproducibility.

4. The quality appraisal demonstrates important weaknesses among included studies.

The authors report that:

No study adequately addressed researcher positioning.

Many studies lacked reflexivity.

Several studies lacked ethical reporting.

Only a minority presented direct participant quotations.

Despite these limitations, all studies were retained in the synthesis.

The manuscript would benefit from:

The author should state the implications of methodological weaknesses for confidence in the findings.

5. The stated objective is to understand the meaning patients express through complaints. However, the quality assessment indicates that only a small number of studies contained substantial participant quotations.

The authors should discuss how this limitation may affect the depth of interpretive claims and the extent to which it reflects patient voices versus author interpretations from primary studies.

6. The description of the thematic synthesis process remains relatively broad.

The manuscript would benefit from additional detail regarding code development, theme generation, management of disagreements between reviewers, and decisions used to prioritize latent versus semantic themes.

This includes examples of coding progression from raw data to final themes that may increase transparency and trustworthiness.

7. There appears to be conceptual overlap between deficient clinical treatment and unethical and unprofessional conduct.

Examples such as informed consent failures, inadequate follow-up care, negligence, and treatment planning concerns could plausibly fit within either category.

The authors should clarify how coding boundaries were established and maintained during analysis.

8. The authors indicate that searches were completed in September 2024. However, several references cited in the Discussion are from 2025 and 2026.

The authors should specify: Whether the searches were updated, whether citation searching continued after database searching, and the final date on which literature was screened.

This is important for transparency and reproducibility.

9. Several statements in the Discussion appear stronger than warranted by the results.

Examples include assertions that patients perceive that their needs are unimportant, complaints represent the culmination of deteriorating trust, and complaints reflect impaired fiduciary relationships.

These interpretations are plausible but should either be more cautiously phrased or more explicitly connected to supporting evidence from the included studies.

10. Reporting of Review Processes

To strengthen methodological transparency, the authors should consider reporting the following:

Reviewer agreement statistics, number of disagreements during title/abstract and full-text screening, procedures used to resolve disagreements, and additional details regarding audit trails and reflexive documentation.

Such information would improve confidence in the review process.

11. Several minor issues should be corrected: Some quotations are excessively long and could be condensed. There are occasional grammatical inconsistencies and awkward wording.

The phrase "the synthesis so derived" could be revised for clarity.

Table 2 may benefit from clearer explanation or simplification.

**********

what does this mean?). If published, this will include your full peer review and any attached files.

If you choose “no”, your identity will remain anonymous but your review may still be made public.

**Do you want your identity to be public for this peer review?**  For information about this choice, including consent withdrawal, please see our Privacy Policy

Reviewer #1: **Yes:**  ِAnfal Mohammad Alqussier

Reviewer #2: **Yes:**  Prof. Patrick Caldicock Ampofo

**********

[NOTE: If reviewer comments were submitted as an attachment file, they will be attached to this email and accessible via the submission site. Please log into your account, locate the manuscript record, and check for the action link "View Attachments". If this link does not appear, there are no attachment files.]

To ensure your figures meet our technical requirements, please review our figure guidelines: https://journals.plos.org/plosone/s/figures

You may also use PLOS’s free figure tool, NAAS, to help you prepare publication quality figures: https://journals.plos.org/plosone/s/figures#loc-tools-for-figure-preparation.

NAAS will assess whether your figures meet our technical requirements by comparing each figure against our figure specifications.

Attachment Submitted filename: review doc.docx

# Author comment: Author response to Decision Letter 1

**DOI:** 10.1371/journal.pone.0362563.r002

6 Oct 2026

RE: Response to reviewer comments

Dear Professor Allam

We appreciate the comments provided in consideration of our article. We have extensively reworked our paper. The following table highlights the responses to the individual reviewer comments.

Editor comments Response

We note that the most recent literature search was completed over a year before the manuscript was submitted to the journal. Systematic and scoping reviews should provide an up-to-date overview of the research topic covered. Therefore, we request that you please update your searches as part of your revision to ensure that the most up-to-date literature is included in your review, or provide scientific justification for not doing so.

Response: We have updated our database search on 26 August 2026 and adjusted the relevant sections in the methodology and results sections accordingly. The PRISMA flow diagram was adjusted accordingly. No new studies were identified for inclusion in this synthesis. The updated search had no impact on the synthesized results or discussion.

Reviewer #1 comments Response

1. Figure 1:

The resolution of Figure 1 (PRISMA flow diagram) is low. Please provide a higher-resolution version to ensure optimal readability in the published version. Response: We have updated the PRISMA flow diagram in a higher-resolution version to ensure optimal readability.

2. Keywords (Line 47):

Please ensure all listed keywords strictly align with standard Medical Subject Headings (MeSH) terms and consider removing overly generic terms. Response: We have changed our keywords to align with standard medical subject headings and removed overly generic terms. Key words were changed from “Patient complaints, oral health, dentistry, dental education, ethics” to “dentistry, qualitative research, patient complaints, meta-synthesis”

3. Introduction:

While the aim of the study is clearly stated, the manuscript would benefit from an explicit and concise definition of the knowledge gap. Additionally, strengthening the theoretical/conceptual framework in the introduction will better highlight the novel contribution of this synthesis.

Response: We have now added a paragraph to the Introduction to explicitly state the knowledge gap and highlight the novel contribution of this synthesis. We also highlighted the interpretive nature of this synthesis.

4. Methods - Search Update (Line 143):

The initial literature search was conducted in September 2024. Please consider updating the search to cover any recent literature published since then, if applicable.

Response: We have updated our database search and adjusted the relevant sections in the Methodology and Results sections accordingly. The PRISMA flow diagram was adjusted accordingly. No new studies were identified for inclusion in this synthesis.

5. Results - Presentation of Direct Quotes:

The Results section relies heavily on extensive direct quotes throughout the narrative text. To improve readability and structural flow, consider condensing these quotes within the main text and transferring extended quotes to a supplementary table or appendix.

Response: We have condensed some of the quotations and removed redundancies in the quotes without compromising the meaning but retaining the qualitative evidence.

6. Discussion - Limitations Section (Lines 578–624):

The Limitations section is currently overly lengthy and somewhat defensive in tone regarding design choices. Please condense this section to focus more concisely on the key methodological and contextual limitations without over-justifying standard qualitative review constraints.

Response: We have condensed the limitations section in a less defensive tone, focusing on the key methodological and contextual limitations. We also added a statement regarding the implications of the methodological weaknesses of the included studies for confidence in our findings.

7. Conclusion (Lines 626–639):

The conclusion effectively synthesizes the main domains; however, it would be considerably strengthened by providing specific, actionable recommendations and practical implications for dental education, clinical practice, and healthcare policy.

Response: We have now added a section “Practical implications and recommendations” to the manuscript and agree that it may considerably strengthen the manuscript and conclusion.

____________________________

Reviewer #2 comments Response

1. The central conclusion that patient complaints represent "breakdowns in the fiduciary patient-practitioner relationship" appears to exceed the evidence presented in the Results section. The authors should demonstrate more explicitly how the primary data support this interpretation and clarify whether this framework emerged from the data or was informed by pre-existing ethical theory.

The author should also consider and discuss alternative explanatory frameworks for patient complaints such as patient expectations, service quality, healthcare accessibility, and patient safety.

Response: We have rephrased the Introduction to highlight the interpretive nature through a relational-ethical lens. In the Discussion section, we qualify this interpretative inference accordingly and describe the coherence this inference provides in explaining the relevant patient complaints.

2. A major methodological concern is the inclusion of highly diverse sources of complaints, including litigation claims, regulatory complaints, online reviews, dental school complaints, and public dental service complaints.

These represent fundamentally different forms of patient feedback with different thresholds, motivations, and contexts. The authors should provide stronger justification for synthesizing these sources as a single higher-order case or provide subgroup comparisons demonstrating thematic consistency across complaint types.

Response: We have now done a subgroup comparison to highlight consistency across subgroups and added the table to the Result section.

3. Fourteen of the nineteen included studies were mixed-methods studies.

The manuscript insufficiently explains:

How qualitative findings were isolated from quantitative findings.

How qualitative data were extracted.

Whether quantitative complaint categories influenced coding and theme development.

Greater transparency regarding data extraction procedures would strengthen methodological rigor and reproducibility.

Response: We have now revised the Methods section to describe the data extraction and coding boundaries in a more explicit and transparent way

4. The quality appraisal demonstrates important weaknesses among included studies.

The authors report that:

No study adequately addressed researcher positioning.

Many studies lacked reflexivity.

Several studies lacked ethical reporting.

Only a minority presented direct participant quotations.

Despite these limitations, all studies were retained in the synthesis.

The manuscript would benefit from:

The author should state the implications of methodological weaknesses for confidence in the findings.

Response: This refers partly to comment 6 of Reviewer#1. We have added a statement in the limitations about the implications of methodological weaknesses of the included studies for confidence in our findings

5. The stated objective is to understand the meaning patients express through complaints. However, the quality assessment indicates that only a small number of studies contained substantial participant quotations.

The authors should discuss how this limitation may affect the depth of interpretive claims and the extent to which it reflects patient voices versus author interpretations from primary studies.

Response: This was stated in the methods as well as the limitations section. We have also qualified the interpretive inference in the introduction and discussion section. To improve transparency in the results we have indicated in table 2 the origin of the data as “primary” or “secondary” in relation to their theme contribution.

6. The description of the thematic synthesis process remains relatively broad.

The manuscript would benefit from additional detail regarding code development, theme generation, management of disagreements between reviewers, and decisions used to prioritize latent versus semantic themes.

This includes examples of coding progression from raw data to final themes that may increase transparency and trustworthiness.

Response: We have revised the Methods section analytic process and have described code progression, theme generation and management of disagreements in a more transparent way.

7. There appears to be conceptual overlap between deficient clinical treatment and unethical and unprofessional conduct.

Examples such as informed consent failures, inadequate follow-up care, negligence, and treatment planning concerns could plausibly fit within either category.

The authors should clarify how coding boundaries were established and maintained during analysis.

Response: We have revised the Methods section to make the analytic procedure more explicit, and we have added examples illustrating how potentially overlapping material was considered during the analytic process. We also describe now that, by the nature of complaints being experiential, imbrication may be expected rather than being categorically discrete; their boundaries represent analytic distinctions developed to capture the emphasis and predominant meaning of patients’ complaints while accepting the interconnectedness of the experiences.

8. The authors indicate that searches were completed in September 2024. However, several references cited in the Discussion are from 2025 and 2026.

The authors should specify: Whether the searches were updated, whether citation searching continued after database searching, and the final date on which literature was screened.

This is important for transparency and reproducibility.

Response: This also refers to comment 4 of Reviewer#1

We have updated our database search and adjusted the relevant sections in the Methods and Results accordingly to display this. The PRISMA flow diagram was also adjusted accordingly. No new studies were identified for inclusion in the synthesis

9. Several statements in the Discussion appear stronger than warranted by the results.

Examples include assertions that patients perceive that their needs are unimportant, complaints represent the culmination of deteriorating trust, and complaints reflect impaired fiduciary relationships.

These interpretations are plausible but should either be more cautiously phrased or more explicitly connected to supporting evidence from the included studies. Response: We have now rephrased the interpretations more cautiously. Also see also our response to comment no 1.

10. Reporting of Review Processes

To strengthen methodological transparency, the authors should consider reporting the following:

Reviewer agreement statistics, number of disagreements during title/abstract and full-text screening, procedures used to resolve disagreements, and additional details regarding audit trails and reflexive documentation.

Such information would improve confidence in the review process.

Response: We have now included a phrase to inform readers that an audit trial of the review process was kept in Covidence, and we have included the reviewer agreement statistics.

11. Several minor issues should be corrected: Some quotations are excessively long and could be condensed. There are occasional grammatical inconsistencies and awkward wording.

The phrase "the synthesis so derived" could be revised for clarity.

Table 2 may benefit from clearer explanation or simplification.

Response: (Also refer to Comment 5 of Reviewer#1.)

We have condensed some of the quotations and removed redundancies in the quotes without compromising the meaning, but retaining the qualitative evidence

The phrase “the synthesis so derived” has been rephrased to “the resulting synthesis”

Table 2 could not be simplified without changing the meaning of the results; additional information was however, added to provide a better understanding and transparency.

Thank you for considering this manuscript.

Sincerely,

Attachment Submitted filename: Response to reviewers.docx

# Peer review: Decision Letter 1

**DOI:** 10.1371/journal.pone.0362563.r003

**Authors:** Eman Allam

28 Oct 2026

Patient complaints in dentistry: A qualitative meta-synthesis

PONE-D-26-35871R1

Dear Dr. Snyman,

We’re pleased to inform you that your manuscript has been judged scientifically suitable for publication and will be formally accepted for publication once it meets all outstanding technical requirements.

Within one week, you’ll receive an e-mail detailing the required amendments. When these have been addressed, you’ll receive a formal acceptance letter and your manuscript will be scheduled for publication.

An invoice will be generated when your article is formally accepted. Please note, if your institution has a publishing partnership with PLOS and your article meets the relevant criteria, all or part of your publication costs will be covered. Please make sure your user information is up-to-date by logging into Editorial Manager at Editorial Manager® and clicking the ‘Update My Information' link at the top of the page. For questions related to billing, please contact billing support.

If your institution or institutions have a press office, please notify them about your upcoming paper to help maximize its impact. If they’ll be preparing press materials, please inform our press team as soon as possible -- no later than 48 hours after receiving the formal acceptance. Your manuscript will remain under strict press embargo until 2 pm Eastern Time on the date of publication. For more information, please contact onepress@plos.org.

Kind regards,

Eman Allam, PhD

Academic Editor

PLOS One

Additional Editor Comments (optional):

Reviewers' comments:

Reviewer's Responses to Questions

**Comments to the Author**

Reviewer #1: All comments have been addressed

Reviewer #2: All comments have been addressed

**********

2. Is the manuscript technically sound, and do the data support the conclusions?

Reviewer #1: Yes

Reviewer #2: Yes

**********

3. Has the statistical analysis been performed appropriately and rigorously?

Reviewer #1: N/A

Reviewer #2: Yes

**********

4. Have the authors made all data underlying the findings in their manuscript fully available?

The PLOS Data policy

Reviewer #1: Yes

Reviewer #2: Yes

**********

5. Is the manuscript presented in an intelligible fashion and written in standard English?

Reviewer #1: Yes

Reviewer #2: Yes

**********

Reviewer #1: I thank the authors for their careful revision; all my previous comments have been satisfactorily addressed.

The manuscript is technically sound, and the conclusions are supported by the data.

I recommend acceptance; minor typographical inconsistencies can be corrected at the proofing stage..

Reviewer #2: The conclusion still states, "Formal complaints may be best understood as indicators of breakdowns in the fiduciary patient-practitioner relationship." The results do not directly demonstrate fiduciary breakdown.

Also, the discussion of patient expectations, service quality, healthcare accessibility, and patient safety were not explicitly discussed as alternative explanations. They appear only indirectly through citations.

All comments have been adequately satisfied.

**********

what does this mean?). If published, this will include your full peer review and any attached files.

If you choose “no”, your identity will remain anonymous but your review may still be made public.

**Do you want your identity to be public for this peer review?**  For information about this choice, including consent withdrawal, please see our Privacy Policy

Reviewer #1: **Yes:**  Dr. Anfal AlQussier

Reviewer #2: **Yes:**  Prof. Patrick Caldicock Ampofo

**********

# Editor report: Acceptance letter

**DOI:** 10.1371/journal.pone.0362563.r004

**Authors:** Eman Allam

PONE-D-26-35871R1

PLOS One

Dear Dr. Snyman,

I'm pleased to inform you that your manuscript has been deemed suitable for publication in PLOS One. Congratulations! Your manuscript is now being handed over to our production team.

At this stage, our production department will prepare your paper for publication. This includes ensuring the following:

* All references, tables, and figures are properly cited

* All relevant supporting information is included in the manuscript submission,

* There are no issues that prevent the paper from being properly typeset

You will receive further instructions from the production team, including instructions on how to review your proof when it is ready. Please keep in mind that we are working through a large volume of accepted articles, so please give us a few days to review your paper and let you know the next and final steps.

Lastly, if your institution or institutions have a press office, please let them know about your upcoming paper now to help maximize its impact. If they'll be preparing press materials, please inform our press team within the next 48 hours. Your manuscript will remain under strict press embargo until 2 pm Eastern Time on the date of publication. For more information, please contact onepress@plos.org.

You will receive an invoice from PLOS for your publication fee after your manuscript has reached the completed accept phase. If you receive an email requesting payment before acceptance or for any other service, this may be a phishing scheme. Learn how to identify phishing emails and protect your accounts at https://explore.plos.org/phishing.

If we can help with anything else, please email us at customercare@plos.org.

Thank you for submitting your work to PLOS One and supporting open access.

Kind regards,

PLOS One Editorial Office Staff

on behalf of

Dr. Eman Allam

Academic Editor

PLOS One
